# Approaches to predict future type 2 diabetes mellitus and chronic kidney disease: A scoping review

**DOI:** 10.1371/journal.pone.0325182

**Published:** 2025-06-11

**Authors:** Anna Bußmann, Christian Speckemeier, Alexandra Ehm, Bettina Kollar, Anja Neumann, Silke Neusser

**Affiliations:** 1 Essener Forschungsinstitut für Medizinmanagement (EsFoMed) GmbH, Essen, Germany; 2 Boehringer Ingelheim International GmbH, Ingelheim am Rhein, Germany; Istituto Di Ricerche Farmacologiche Mario Negri, ITALY

## Abstract

**Background:**

Demographic change and changing lifestyles are leading to a steady increase in so-called population diseases such as type 2 diabetes mellitus and chronic kidney disease. Both conditions are often preceded by a latency period during which lifestyle changes and/or medications have the potential to delay or even prevent disease onset. Thus, detection of those at an increased risk of these diseases is of great importance. A scoping review was conducted to collate different prediction approaches for type 2 diabetes mellitus and chronic kidney disease.

**Methods:**

Literature searches were performed in PubMed, Embase, Web of Science, and Google Scholar. A stepwise approach was used, consisting of searches for systematic reviews and primary literature, and additional Google searches for novel approaches. Included was literature that (1) presented an approach for risk prediction of incident type 2 diabetes mellitus or chronic kidney disease, (2) contained information on the risk factors considered and application, (3) targeted the general population, (4) was written in English or German language, and (5) for which an abstract and full-text was available. Literature screening was carried out by two persons independently.

**Results:**

Studies extracted literature from 1940 to 2023. Prediction approaches were included from 25 literature reviews, eight primary studies and nine studies found in additional searches. Several different approaches were identified, including methods based on clinical parameters, biological parameters (blood, urine, microbiome, genetics), the combinations of those, sequential approaches, and exposure and lifestyle factors. Most of the identified approaches were risk surveys that usually ask for simple and readily available parameters. Novel approaches cover transdermal optical imaging, prediction based on facial blood flow and using deoxyribonucleic acid methylation data.

**Conclusion:**

This scoping review provides an overview of different tools for the risk prediction of type 2 diabetes mellitus and chronic kidney disease. In addition to established tools, which are primarily risk surveys, innovative approaches have been developed and evaluated in recent years in which the potential of machine learning is utilized. As cardio-renal-metabolic diseases share predicting factors and given the social and economic importance of these diseases, approaches that address multiple relevant diseases such as type 2 diabetes mellitus, chronic kidney disease and cardiovascular disease can be of great interest, especially in time- and resource-constrained healthcare settings.

## Introduction

Demographic shifts and changing lifestyles contribute to a steady increase in so-called population diseases [1]. These include type 2 diabetes mellitus (T2DM) and chronic kidney disease (CKD), among others [2]. Due to complex interrelationships between these diseases, T2DM and CKD often occur together with heart failure (HF) [3,4]. Insights from various research highlight the interconnection between T2DM, HF, and CKD, termed cardio-renal-metabolic (CRM) disease [5–7]. Because of these multidirectional interactions, the growing incidence of T2DM is accompanied by an increasing prevalence of both HF and CKD [6].

T2DM, which accounts for 90–95% of patients with diabetes [8,9], is a complex disease characterized by hyperglycemia, resulting from defects in insulin secretion and/or action [10]. Diabetes causes microvascular and macrovascular damage, associated with an increased risk of stroke, neuropathy, cardiovascular disease, retinopathy, renal disease, and amputation [11]. Globally, about 529 million individuals were affected by T2DM in 2021 [12]. Projections indicate an increase of this number by 46% to 783 million by 2045 [13]. Increase in prevalence is mainly due to the rise in obesity, sedentary lifestyles and energy-dense diets, as well as demographic change [9,10]. Globally, expenditures due to diabetes are estimated to account for 11.5% of total health spending [14], with the financial impact being projected to increase in the coming years [15]. An examination of a US-based outpatient registry found that only around 6% of patients with T2DM had isolated T2DM without other CRM conditions, while around half of T2DM patients suffered from three or more other CRM conditions [16], with the most prevalent conditions being hypertension, hyperlipidemia, coronary artery disease and CKD. T2DM causes adaptive hyperfiltration in the kidneys, which leads to long term damage of functioning nephrons [17]. As a result, CKD is commonly referred to as diabetic kidney disease (DKD) [18]. Across Europe, around 100 million individuals suffer from CKD and cause costs of an estimated € 140 billion annually. According to projections, CKD will be the fifth leading cause of death by 2040 globally [19]. In total, more than one billion people suffer from HF, CKD and T2DM worldwide [20] and these diseases together are among the leading causes of death, with 20 million deaths per year [21,22].

Studies investigating the trajectories of disease onset have found that diagnosis of T2DM is preceded by a latency period of around ten years [23,24]. It is estimated that for every person diagnosed with diabetes, there is one undiagnosed person [25]. In addition, it is estimated that around one third of the US population had prediabetes in 2015 and of these, 90% were not aware of their condition [26]. During this period, lifestyle changes, pharmaceuticals or both have the potential to delay or even reverse onset of T2DM [9]. Also in CKD, the gradual loss of kidney function usually remains unnoticed for many years [27]. A study in Germany concluded that female patients in particular were unaware of their CKD disease, regardless of CKD stage [28]. Preventive measures at this stage can include glucose-lowering medications and blood pressure control [29]. In light of the health and financial impact imposed by T2DM and CKD, early detection of those at an increased risk of these diseases is of great importance. Identifying persons being at risk for developing T2DM or CKD in the future is considered essential for prevention and early action [27,30]. A number of studies have illustrated the value of early identification of people at increased risk for disease or manifest disease and the value of timely interventions such as referral and participation in lifestyle change programs and care programs [31–33]. Because of the strong societal and economic impact of these two diseases and the great potential of prediction, it is important to identify and describe the different prediction approaches. Accordingly, the aim of this scoping review is to provide an overview of different prediction approaches for T2DM and CKD.

## Methods

The conduction of the scoping review followed the five key phases of the Arksey and O’Malley framework outlined by Levac et al.: (1) identifying the research question, (2) identifying relevant studies, (3) study selection, (4) charting the data, and (5) collating, summarizing, and reporting the results [34]. Conduct of this review was guided by the Joanna Briggs Institute’s (JBI) guide for scoping reviews [35] and reporting followed the Preferred Reporting Items for Systematic Reviews and Meta-Analyses (PRISMA) extension for scoping reviews [36]. A review protocol was written and registered with the Open Science Framework (OSF, https://osf.io/vrfta/) prior to full-text screening of identified systematic reviews [37]. The review aims to capture different approaches according to broad categories (e.g., clinical predictors, biological predictors) and their intended use, rather than to fully map each approach with the totality of all its associated tools. Thus, the following results do not represent whether these approaches are regularly applied by public health or patient care providers.

### Search strategy

Literature searches were performed in a stepwise approach. In the first stage, only systematic reviews were included. In a second step, literature searches for primary literature containing original research data were conducted regarding the approaches that have been found in the first stage but did not match the inclusion criteria and were therefore not included in step one. In a third step, potential future prediction approaches or those under development that have not been covered in the primary literature were searched.

[1] The search strategy for systematic reviews included the terms “prediction”, “approach” and “T2DM”/”CKD” and respective synonyms. The search terms were further specified using keywords from the relevant databases, such as Medical Subject Headings (MeSH). The electronic indexed databases PubMed (1946 – present), EMBASE (1947 – present) and Web of Science (1900 – present), as well as Google Scholar were searched.
[2] The search strategy for primary literature was outlined which included the terms “prediction”, “approach”, “T2DM”/ ”CKD” and the corresponding approaches that have been found in step one but did not meet the inclusion criteria of the first search. Again, synonyms and specified keywords from the relevant databases were used. The searches were conducted in the same databases. Corresponding to the time horizons of searches within the reviews, a filter was set within the search strategy to include literature which was published after the corresponding review. Both strategies were pilot tested in the databases and refined in two rounds. The full search strategies and number of hits per keyword for step one and two are listed in Appendices S1 and S2.
[3] Additional searches in Google Scholar and on websites of academic organizations, medical societies, private businesses, and industry were conducted using keywords and synonyms for “prediction” and “T2DM”/”CKD” aiming to cover potential future prediction approaches or novel approaches under development that have not been covered in step one and two.

### Eligibility criteria

Studies with the following inclusion criteria were eligible in all three steps: (1) presented an approach for risk prediction of incident T2DM or CKD (hence, before onset of disease), (2) contained information on the risk factors considered and application (e.g., self-assessment survey, test conducted in pharmacies, …), (3) targeted the general population, (4) written in English or German language, and (5) for which an abstract and full-text was available. For CKD, approaches were also included in which T2DM is included as an existing disease, as T2DM is an important predictive parameter.

Studies were excluded if they met the following criteria: (1) Studies that did not include a prediction tool, (2) studies without information on the risk factors considered within the prediction tools, (3) studies presenting a prognostic approach for T2DM or CKD after disease onset, (4) studies focusing on prediction tools that apply only to a specific population and/or specific comorbidities (excluding T2DM for the approaches predicting CKD), (5) studies not in English or German language, (6) studies with no full-text available.

### Screening and extraction

The literature results were downloaded into the EndNote reference management program. Duplicates were removed automatically and manually during the screening process. All unique references were screened in terms of their potential relevance based on title and abstract. Screening was based on screening forms which were pre-tested and revised. Documents considered potentially relevant were reviewed in full-text and retained if the study met inclusion criteria. Two researchers performed all screening steps independently. Any disagreements were resolved by consulting a third person. Results were extracted into pre-specified tables comprised of the following aspects: first author, year of publication, name of tool, country, and parameters contained in prediction approach. The methodological quality of the included documents was not assessed as the focus of this review was to present an overview of prediction methods. The evidence underlying the recommendations was not reviewed.

## Results

### T2DM

**Fig 1:**
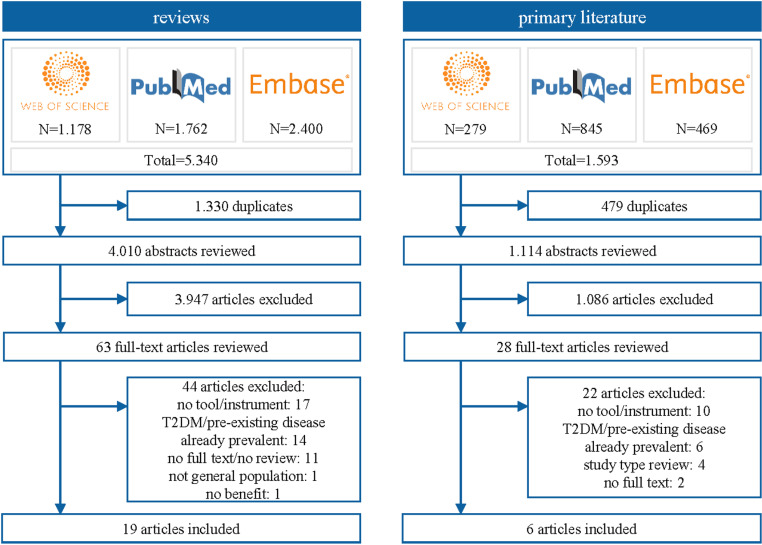
Flow chart of the literature searches for T2DM. Note: Excluded full-texts may fulfil more than one exclusion criterion.

[1] In the first step, a total of 5,340 systematic reviews were identified. After removal of duplicates 4,010 articles underwent title and abstract screening, with 63 articles being reviewed in full-text. A total of 19 systematic reviews met the inclusion criteria [25,38–55]. Four reviews found in step one did not meet the inclusion criteria but addressed further approaches that have not been covered in step one. These included branched chained amino-acids (BCAA) as a predictor, the protein fetuin-A, microbiota and continuous glucose monitoring (CGM). An overview of the studies can be found in Appendix S3.
[2] For these approaches, primary literature was searched for in the second step. The search identified 1,593 articles. After removal of duplicates, 1,114 articles underwent title and abstract screening, 28 articles were reviewed in full-text and six publications were included [30,56–60]. No studies could be found concerning the use of CGM or fetuin-A for prediction of T2DM.
  The selection process of the searches for systematic reviews in step one and primary studies in step two is shown in Fig 1.
[3] The third step of targeted searches for additional promising approaches identified an additional four prediction approaches. Study characteristics of the included studies of the three steps can be found in Appendix S4.

Table 1 shows different categories of approaches for prediction of T2DM identified and a number of examples for these approaches. Appendix S7 provides information on sample size of the studies reporting on the identified tools, validation of the approaches, and application in clinical practice. In brief, sample size ranged between 608 and 359,349 in studies providing prediction approaches for T2DM. Validation studies have been found for 17 of the 21 T2DM prediction approaches. For some of the prediction approaches, updates have been found. Many of the tools were reported to be actively used for prediction; however, for a number of tools, no information on usage could be found.

**Table 1:** Prediction categories for T2DM differentiated by intended use and examples for tools within the categories.

Category	Intended use	Example tools	Brief description/ Data	Found in
Biological predictor/ Identification of impaired glucose tolerance	Test to be conducted at a physician or laboratory	Oral glucose tolerance test [61] USA	Used to diagnose impaired glucose tolerance. 2-hour oral glucose tolerance test: fasting glucose will be drawn and two more blood samples need to be taken after drinking a glucose-containing liquid.	1 [38,47,53]
Clinical predictor/ Survey tool	Self-assessment survey, results in a score between 0 and 20 points	FINDRISC^a^ [62] Finnland	Survey aims to predict risk of developing T2DM in the next ten years. Contains age, waist circumference, BMI, BP, physical inactivity, eating habits.	1 [38,41,43,46,47]
Clinical predictor/ Survey tool	Self-assessment survey, results in low risk, moderate risk, and high risk	CANRISK^b^ [63] Canada	Survey aims to predict risk of having pre-diabetes or T2DM. Contains age, sex, BMI, waist circumference, physical inactivity, history of high BP & blood glucose & gestational diabetes, family history, ethnicity, education.	1 [43]
Clinical predictor/ Survey tool	Self-assessment survey, results in low risk (1 in 100 will develop T2DM), intermediate risk (1 in 50), and high risk (1 in 3)	AUSDRISK^c^ [64] Australia	Survey aims to predict the risk to develop T2DM. Contains age, sex, ethnicity, family history, history of high blood glucose, drugs for high BP, smoking status, eating habits, physical inactivity, waist circumference.	1 [46,47]
Clinical predictor/ Survey tool	Self-assessment survey, results in advice for future lifestyle changes	German Diabetes risk score^d^ [65] Germany	Survey aims to predict the risk to develop T2DM in the next ten years. Contains sex, age, history of high BP, height, waist circumference, family history, eating habits, drinking habits (coffee), physical inactivity, smoking status.	1 [46,55]
Clinical predictor/ Survey tool	Unclear *Population-based risk prediction tool to understand diabetes risk in population and to inform heath policy* [66]	DPoRT [66] Canada	Tool estimates the number of individuals who will develop physician-diagnosed diabetes. Contains BMI, age, ethnicity, hypertension, immigrant status, smoking status, education, heart disease.	1 [47]
Clinical predictor/ Survey tool	Unclear *Based on variables stored in patients’ health records, or which patients are likely to know* [42]	QDScore [67] UK	Survey aims to predict the risk to develop T2DM in the next ten years. Contains age, sex, BP, BMI, family history, smoking status, ethnicity, cardiovascular disease, Townsend deprivation score, current use of corticosteroids.	1 [42,52]
Clinical predictor/ survey tool	Self-assessment survey, results in advice for lifestyle modifications	Thai Diabetes risk score [68] Thailand	diabetes risk scoring system for identifying individuals who are likely to develop diabetes in the near future. Contains BMI, age, sex, waist circumference, history of high BP, family history.	1 [42,44,46,53,55]
Clinical + biological predictors	Multivariable predictive function as an alternative to OGTT	Diabetes score based on ARIC study [31] USA	Function to predict future occurrence of T2DM. Contains waist circumference, height, hypertension, BP, family history, ethnicity, age, fasting glucose, triglycerides levels, HDL-cholesterol.	1 [38,42,46,47,55]
Clinical + biological predictors	Self-calculation based on national regular health screening tests	Korean Diabetes risk score [69] Korea	Score to identify individuals at high risk of developing T2DM. Contains age, family history, smoking status, physical inactivity, use of antihypertensive therapy, use of statin therapy, BMI, BP, total cholesterol, fasting glucose, γ-glutamyltransferase (only in women), drinking habits (only in men).	1 [39]
Clinical + biological predictors	Variables which are ordinarily available in routine clinical setting, intended to be an alternative to OGTT	San Antonio study prediction model [70] USA	Score to identify individuals at risk of developing T2DM in 7.5 years. Contains medical history, BMI, BP, fasting glucose, fasting serum total, LDL-cholesterol, HDL-cholesterol and triglycerides levels.	1 [38,41,47,51]
Clinical + biological predictors	Contains simple parameters which are available at a clinic visit with a physician.	Framingham Offspring study Type 2 diabetes prediction model [71] USA	Prediction algorithm aims to estimate risk of new T2DM in the next 7 years. Contains family history, obesity, hypertension, HDL-cholesterol, triglycerides level, fasting glucose	1 [41]
Clinical + biological predictors	Unclear Sex-specific prediction parameters	Prediction score based on DESIR study [72] France	Simple clinical risk score to predict later T2DM. Men: fasting glucose, smoking status, waist circumference, γ-glutamyltransferase Women: fasting glucose, BMI, family history, triglycerides levels.	1 [38,41,55]
Clinical + biological predictors	Calculation of visceral fat	Visceral Adiposity Index [73] Italy	Simple formula which estimates the accumulation of visceral fat, which can be used as a predictor of T2DM. Contains waist circumference, triglycerides levels, HDL- cholesterol.	1 [48]
Clinical + biological predictors/ Sequential approach	Concept for community pharmacy setting, Individuals are referred to general practitioner or receive advice on lifestyle changes.	No name [74] Switzerland	Sequential screening concept to identify previously unidentified persons with or at high risk of T2DM. Consists of - ADA risk assessment questionnaire [75] to be filled out by the individuals themselves, - in individuals potentially at-risk, a set of six risk factors was assessed by the pharmacy team (contains age, BMI, history of high BP, family history, physical inactivity, history of delivering a baby over 4 kg (women only)) - at-risk individuals further received measurement of capillary blood glucose by pharmacist.	1 [52]
Biological predictors/ genetic biomarker	Calculation of genetic risk score	Genotype score [76] USA	Genotype score to predict diabetes risk in the community. Using 18 single-nucleotide polymorphisms, a genotype score ranging from 0 to 36 was constructed on the basis of the number of risk alleles.	1 [49]
Exposure + lifestyle factors	Combination of multiple self-reported non-genetic exposure and lifestyle factors	Polyexposure score, [77] USA	Machine learning model to select the most predictive and robust factors for developing prospective T2DM. Contained 111 exposure and lifestyle factors recorded in the UK Biobank (UKB). 12 variables (containing factors that indicated drinking habits, eating habits, early life factors, household information, sleeping habits, smoking status). were selected for the final model using a lasso-based method that relied on summary statistics.	1 [25]
Clinical + biological predictors/ Electronic medical records	Deep Learning approach based on previous illness history from electronic medical records	DeepCare [78] Australia	Model predicts future medical outcomes. Electronic medical records from planned and unplanned admissions, containing lab tests, diagnoses, procedures, medications, and clinical narratives.	1 [25]
Clinical + biological predictors/ survey + gut microbiome biomarkers	Machine learning approach based on stool samples	No Name [56] Denmark	Gut microbiome markers were identified, which provide a predictive measure for metabolic traits related to T2DM. Approach provides additional variables for personal risk assessment.	2
Smartphone app	User makes a 30-second video selfie	NuraLogix Corp. [79] Canada	Smartphone app aims to predict the risk for pre-diabetes. App uses a novel imaging method called transdermal optical imaging.	3
Clinical & biological predictors/ genetic biomarker	Unclear	University of Edinburgh [80] UK	Prediction of the ten-year risk of developing T2DM by inclusion of DNA methylation data. DNA test alongside classical risk factors, such as sex, age, and BMI.	3

ADA: American Diabetes Association; ARIC study: Atherosclerosis Risk in Communities study; BMI: body mass index; BP: blood pressure; DESIR: Data from the Epidemiological Study on the Insulin Resistance Syndrome; DNA: deoxyribonucleic acid; DPoRT: Diabetes Population Risk Tool; HDL: high-density lipoprotein; LDL: low-density-lipoprotein; OGTT: oral glucose tolerance test; T2DM: type 2 diabetes mellitus; UKB: UK Biobank.

1: identified in systematic reviews; 2: identified in primary literature; 3: identified in structured search.

a FINDRISC (Finnish Diabetes Risk Score; https://www.mdcalc.com/calc/4000/findrisc-finnish-diabetes-risk-score).

b CANRISK (Canadian Diabetes Risk Questionnaire; https://health.canada.ca/apps/canrisk-standalone/pdf/canrisk-en.pdf).

c AUSDRISK (Australian Type 2 Diabetes Risk Assessment Tool; https://www.health.gov.au/sites/default/files/the-australian-type-2-diabetes-risk-assessment-tool-ausdrisk.pdf).

d German Diabetes risk score (https://drs.dife.de/).

As described in the methods section, this scoping review does not list all identified predictive approaches but provides examples of tools in each of the delineated categories and areas of application.

**Fig 2:**
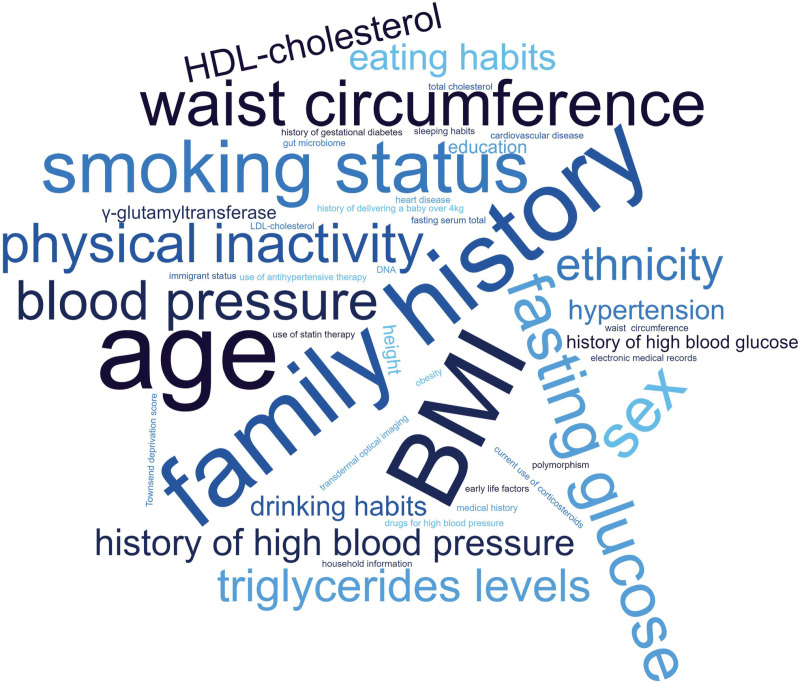
Word cloud of the occurrences of different prediction parameters for T2DM.

An overview of the occurrences of different prediction parameters used in the identified tools of Table 1 is demonstrated in Fig 2. The size of words in the word cloud reflects the frequency of appearance in the prediction tools.

In the following, the prediction categories and exemplary approaches identified in the three research steps are presented. The 2-hour oral glucose tolerance test (OGTT) has been identified as the classic means for biological predictors to identify individuals at increased risk for future T2DM. Impaired glucose tolerance is identified by taking blood samples before and after drinking a glucose-containing liquid [61]. OGTT is usually taken as a solitary measure, i.e., without taking other risk factors into account [70].

A large number of risk surveys was identified. Typically, these surveys ask for clinical risk factors such as age, family history of diabetes, and body mass index (BMI), among others. Many of the validated tools are country specific self-assessment scores, such as the Finnish Diabetes Risk Score (FINDRISC), the Canadian Diabetes Risk Questionnaire (CANRISK), the Australian Type 2 Diabetes Risk Assessment Tool (AUSDRISK) and the German Diabetes risk score. Other common tools include the American Diabetes Association (ADA) Risk Tool [81], the Indian Diabetes risk score (IDRS) [82], the Thai Diabetes risk score [68] and the Diabetes Risk Calculator based on the Third National Health and Nutrition Examination Survey (NHANES) [83]. Some surveys are based on data from patients’ health records [67].

Other prediction approaches involving biological parameters contain parameters such as high-density lipoprotein (HDL)-cholesterol, triglycerides levels, or fasting glucose in addition to clinical parameters. Information on the biological parameters is either readily available to the individual [69] or might necessitate a physician visit [71]. For example, the Korean Diabetes Risk Score aims to identify persons at high risk of developing T2DM in the next ten years based on national regular health screening tests in Korea. Thus, the general population may calculate their risk score without any additional examinations [69]. Another example is the San Antonio Study Prediction Model [70], which includes information on age, sex, ethnicity, as well as readily available clinical measurements such as systolic blood pressure, BMI, and family history of diabetes as well as biological measurements like HDL-cholesterol and impaired fasting plasma glucose [84]. In other (sequential) approaches, an initial testing is carried out in the pharmacy [74,85] or in dental practices [86,87] and in case of abnormal values, a referral is made. Some approaches, such as the Data from the Epidemiological Study on the Insulin Resistance Syndrome (DESIR) approach [72], strongly take into account sex-specific differences in the choice of clinical and biological parameters.

In the included studies, additional potential predicting markers have been investigated, such as amino acids (AA) [30], BCAA [57,60], or lipoproteins [58,59]. For example, Chen et al. verified a strong correlation between BCAA and aromatic amino acids (AAA) with insulin resistance and thus with possible development of T2DM [57]. Another study by Chen et al. added tryptophan levels to the existing AA markers and found better predictive performance for the combined score for incident T2DM [30]. Flores-Guerrero et al. added lipoprotein measurements to the Framingham Offspring prediction algorithm [71], which significantly increased the performance of the original algorithm [88]. One included systematic review investigated whether calculation of the Visceral Adiposity Index (VAI) [73] can predict T2DM in Asian populations [48].

Three of the included systematic reviews deal with machine and deep learning techniques to predict T2DM in hospital, clinical and/or community settings [25,45,50]. For example, DeepCare aims to predict future T2DM based on previous illness history from electronic medical records [78]. Nawi et al. explored the performance of machine learning algorithms in detecting prediabetes such as a prediabetes score proposed by Quan et al. comprising 13 outcomes, including mortality, micro- and macrovascular complications, and the development of T2DM [45,89]. In their systematic review, Silva et al. investigated the performance of machine learning models for prediction of T2DM in the community [50].

In recent years, there has been increased research on the potential application of microbiome in the prediction of T2DM. For example, Aasmets et al. describe a machine learning approach based on stool samples, which provide additional parameters when assessing the personal risk of T2DM [56].

One systematic review [49] investigated polygenic risk scores and included a number of longitudinal studies, such as the genotype score for T2DM [76], which was based on 18 risk alleles and predicted relative risk of diabetes per allele. In addition, He et al. have investigated a polyexposure score based on non-genetic exposure and lifestyle factors which aims to detect incident diabetes [77]. The score includes 12 non-genetic exposure factors such as alcohol intake, diet, early life factors, household information, sleep, and smoking habits.

A number of promising approaches was identified in additional searches. NuraLogix Corp has developed a smartphone app which uses facial blood flow patterns and predicts pre-diabetes risk with a 30-second video selfie. The machine learning-based approach uses a novel imaging method called transdermal optical imaging (TOI) [79] to analyze key features in faces that, in combination with vital signs such as respiration and heart rate, could be a risk predictor for prediabetes. Intelligent Bio solutions (IBS) has developed a non-invasive, saliva-based glucose test [90]. The sensor was developed as a point of care self-test for diabetes for potential future use. Researchers of the University of Edinburgh were able to predict the ten-year risk of T2DM by including DNA methylation (DNAm) data to a classical risk assessment with parameters such as sex, age and BMI more accurately [80,91]. Another approach was suggested by researchers from the School of Public Health at the University of Minnesota. They found links between the composition of subgingival bacteria and changes in future glucose levels. The resulting microbial ‘dysbiosis score’ was found to be a stronger predictor of rising glucose levels than either age or obesity [92].

### CKD

**Fig 3:**
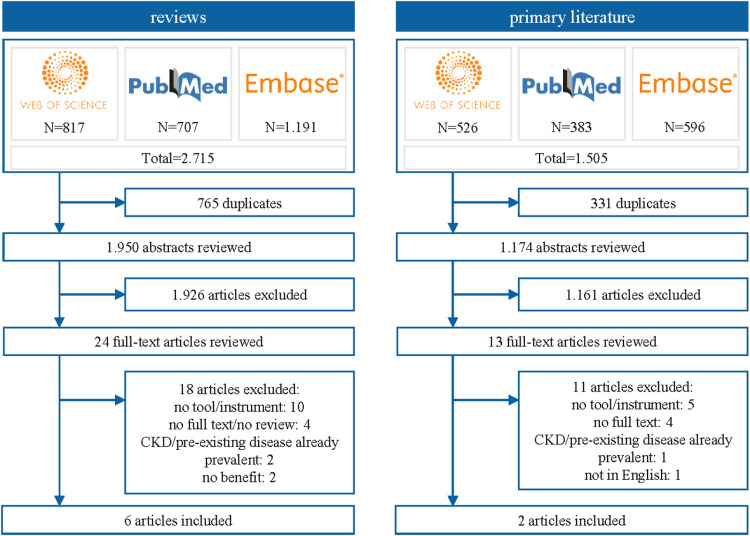
Flow chart of the literature searches for CKD.

[1] In the first step, a total of 2,715 systematic reviews for CKD were identified. After duplicate removal, 1,950 articles were reviewed in title and abstract screening. Twenty-four articles were reviewed in full-text, and six articles met inclusion criteria [27,29,93–96]. Three reviews found in step one did not meet the inclusion criteria but addressed further approaches that have not been covered in step one. These approaches included consideration of Klotho levels, Artificial Intelligence (AI) based on ocular images and glycated hemoglobin (HbA1c) variability. An overview can be found in Appendix S5.
[2] For these three approaches, searches for primary literature were conducted in step two, with 1.174 articles undergoing title and abstract screening. Of these, 13 articles were reviewed in full-text, and two of them were included that address HbA1c in CKD risk prediction [97,98]. No studies could be found concerning the use of Klotho levels and ocular images. The selection process of the literature searches of step one and two is shown in Fig 3.
[3] In step three, five additional studies were identified by targeted searches [99–103]. Characteristics of the identified literature in the three steps can be found in Appendix S6.

**Table 2:** Prediction categories for CKD differentiated by intended use and examples for tools within the categories.

Category	Intended use	Example tools	Brief description/data	Found in
Clinical predictors / survey tool	Online risk calculator for self-assessment^a^, possible use at population level for risk stratification to systematically identify patients who need further investigation	QKidney score [104] UK	Web calculator to evaluate the risk of developing moderate to severe kidney disease over the next five years. Contains: age, sex, ethnicity, smoking status, DM, heart failure, PVD, high blood pressure requiring treatment, rheumatoid arthritis, systemic lupus erythematosus, history of heart attack, angina, stroke or TIA, history of kidney stones, family history of kidney diseases, BMI, SBP.	1 [27,29,95]
Clinical predictors / survey tool	Questionnaire for general practitioners as part of a prevention guideline in the Netherlands to identify people at risk for chronic cardiometabolic disease	Rotterdam-Hoorn score [105] Netherlands	Nonlaboratory-based risk tool to predict CVD, T2DM, and/or CKD. Contains: age, smoking status, use of antihypertensives, use of lipid-lowering medication, BMI, waist circumference, parent or sibling with myocardial infarction or stroke (before the age of 65 years), parent or sibling with DM, history of gestational diabetes.	1 [27]
Clinical predictors / survey tool	Self-assessment questionnaire	SCORED score [106] USA	Score from ≤1 to ≥9 to assess the probability of chronic kidney disease. Contains: age, sex, anemia, BP, DM, history of heart attack or stroke, history of congestive heart failure or heart failure, circulation disease in legs, urine protein.	1 [27,95]
Clinical predictors / survey tool	Point system for clinical practice aiming to screen the general population	Clinical model [107] Taiwan/China	Point-based model from 1 to 17 points using clinical parameters to predict the 4-year incidence of chronic kidney disease. Contains: age, BMI, DBP, history of T2DM, history of stroke.	1 [27,29,95]
Clinical predictors / survey tool	Population-based intervention; applies primarily to groups defined by a parsimonious set of clinically relevant variables rather than directly to individuals; potentials also in clinical practice	Simplified categorial model based on ARIC/CHS [108] USA	Simple scoring algorithm from ≤1 to ≥8 points that stratifies persons at risk of developing clinically significant CKD based on ARIC and CHS study data. Contains: age, female sex, anemia, hypertension, DM, history of CVD, history of heart failure, PVD.	1 [27,29,95]
Clinical + biological predictors / survey tool + blood biomarker	Applied by physicians	PromarkerD [103] Australia	Blood test that measures a panel of three novel plasma biomarkers (ApoA4, CD5L, IGFBP3) combined with three clinical factors (age, HDL-cholesterol, eGFR) to identify patients at risk of developing DKD within the next four years. The combined raw data are submitted to the PromarkerD Hub to generate a test report.	3
Clinical + biological predictors / survey tool + blood biomarker	Online App to assist clinicians to predict the DN risk and to use for community-based DN prevention^b^	Dynamic DN incidence nomogram [100] China	Nomogram to assess the risk of DN incidence in T2DM patients using 8 characteristics. Contains: SBP, DBP, fasting blood glucose, HbA1c, total triglycerides levels, serum creatinine, blood urea nitrogen, BMI.	3
Clinical + biological predictors / survey tool + blood biomarker	Population-based intervention; applies primarily to groups defined by a parsimonious set of clinically relevant variables rather than directly to individuals; potentials also in clinical practice	Best fitting categorial model based on ARIC/CHS [108] USA	Simple scoring algorithm from ≤1 to ≥8 points that stratifies persons at risk of developing clinically significant CKD based on ARIC and CHS study data. Contains: age, white race/ethnicity, female sex, anemia, hypertension, DM, history of CVD, history of heart failure, HDL-cholesterol, PVD.	1 [27,29,94]
Clinical + biological predictors / survey tool + blood biomarker	unclear	HGI risk score [97] China	Combined risk score based on HGI to predict a person’s risk of DKD. Contains: HGI (=observed HbA1c – predicted HbA1c), age, T2DM duration, SBP, total cholesterol, fasting C-Peptide, eGFR.	2
Biological predictors / urinalysis	unclear	DNlite-IVD103 [99] Taiwan	Urinary ELISA test that detects a post translational modified fragment of Fetuin-A to predict short-term renal function change (such as eGFR decline) in T2DM with microalbuminuria.	3 Conference abstract
Clinical + biological predictors / survey tool + urinalysis	Potential use by general public and health care providers to be used for targeted screenings, to electronically check for the presence of the variables in patients data and as self-assessment checklist to estimate the risk of having CKD	Korean Risk Score [109] Korea	Risk prediction algorithm developed using a score from 0 to 10 weighted towards common noninvasive variables. Contains: age, female sex, anemia, hypertension, DM, CVD, proteinuria.	1 [27,95]
Clinical + biological predictors / survey tool + urinalysis	Potential use by community health worker as a two-step targeted screening strategy to easily identify persons who need confirmatory kidney disease testing in India	Point-of-care CKD screening – Model 3a [110] India	Simple point-of-care urine dipstick screening strategy for likelihood of CKD. Contains: age, sex, waist circumference, BMI, SBP, DBP, urine dipstick glucose, urine dipstick albumin.	1 [93]
Clinical + biological predictors / survey tool + blood biomarker + urinalysis	Clinical use of a score chart in which predictors are divided into clinically useful categories	Renal risk score based on the PREVEND study [111] Netherlands	Renal risk score from ≤15 to ≥41 points for the general population to identify individuals at risk for progressive CKD. Contains: age, eGFR, UAE, CRP, SBP, hypertension.	1 [27,94]
Clinical + biological predictors / survey tool + blood biomarker + urinalysis	unclear	Quality-of-care scoring system [101] Taiwan	Quality-of-care scoring system from 0 to 8 points to predict the occurrence of CKD in T2DM patients by combining process indicators (frequencies of HbA1c, lipid profile, urine, foot, and retinal examinations), intermediate outcome indicators (LDL, BP, HbA1c), and comorbidity of hypertension	3
Clinical + biological predictors / survey tool + blood biomarker + urinalysis	Potential use for physicians to determine an individual’s estimated CKD risk and for clinical counseling and decision-making at a primary care level.	Risk score based on the Framingham Heart Study [112] USA	Risk prediction score from 0 to 15 points based on the Framingham Heart Study cohort that estimates an individual’s 10-year probability of developing CKD by using clinical factors readily accessible in primary care. Contains: age, hypertension, DM, baseline eGFR and albuminuria.	1 [27,29,95]
Clinical + biological predictors / survey tool + blood biomarker + urinalysis	Point system for clinical practice aiming to screen the general population	Biochemical model [107] Taiwan/China	Point-based model from 0 to 23 points using clinical + biochemical parameters to predict the 4-year incidence of chronic kidney disease. Contains: age, DBP, history of stroke, uric acid, postprandial glucose, HbA1c, urine protein.	1 [27,94]
Clinical + biological predictors / survey tool + genetic biomarker	unclear	Multifactorial genetic model [113] Israel	Multifactorial logistic regression model to predict nephropathy in DM patients with a total of 14 variables: 5 SNPs accounting for 9 variables and 5 conventional nephropathy predictors: DM, sex, age, duration of diabetes and ethnicity.	1 [27,96]
Clinical + biological predictors / survey tool + blood biomarker + genetic biomarker	unclear	Genetic risk score based on the GWAS study [114] China	Genetic risk prediction model for DN in T2DM patients including traditional risk factors. Contains: age, obesity, sex, abnormal triglycerides levels, hypertension, heart disease and 7 SNPs.	1 [96]
Biological predictors / genetic biomarker	No use as the genetic risk score did not substantially improve disease discrimination beyond clinical risk factors alone.	Genetic risk score based to on the Framingham Heart Study [102] USA	Genetic risk score based to on the Framingham Heart Study cohort including 53 risk alleles to predict incident cases of stage 3 CKD.	3

ADVANCE: Action in Diabetes and Vascular Disease: Preterax and Diamicron MR Controlled Evaluation; ApoA4: apolipoprotein A-IV; ARIC study: Atherosclerosis Risk in Communities study; BP: blood pressure; CD5L: CD5 antigen-like; CKD: chronic kidney disease; CRP: C-reactive protein; CHS: cardiovascular health study; CVD: cardiovascular disease, DBP: diastolic blood pressure; DKD: diabetic kidney disease; DM: diabetes mellitus, DN: diabetic nephropathy; eGFR: estimated glomerular filtration rate; ELISA: enzyme-linked immunoassay test; GWAS: genome-wide association study; HbA1c: glycated hemoglobin; HDL: high-density lipoprotein, HGI: hemoglobin glycation index; IGFBP3: insulin-like growth factor-binding protein 3; LDL: low-density-lipoprotein; PREVEND: Prevention of Renal and Vascular End-stage Disease; PVD: Peripheral vascular disease, SBP: systolic blood pressure; SCORED: Screening for Occult Renal Disease; SNPs: single nucleotide polymorphisms, T2DM: type 2 diabetes mellitus; TIA: transient ischaemic attack; UAE: urinary albumin excretion.

1: identified in systematic reviews; 2: identified in primary literature; 3: identified in structured search.

a QKidney score (https://qkidney.org/).

b DN_Dynamic Monogram (https://doctorhu.shinyapps.io/DN_DynNomapp/).

**Fig 4:**
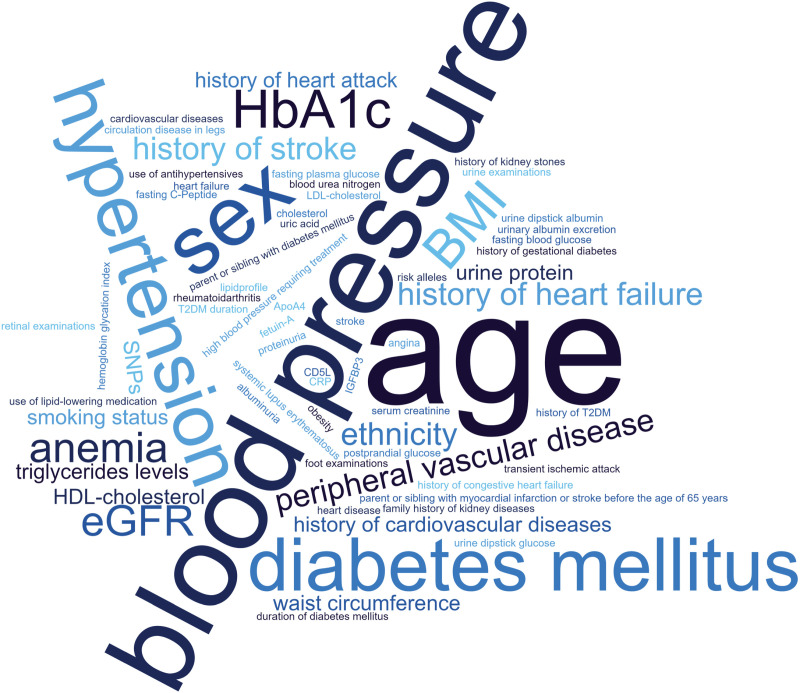
Word cloud of occurrences of different prediction parameters for CKD.

A variety of different risk prediction approaches for the onset of CKD were identified. Those include nonlaboratory clinical predictors, biological predictors such as blood and urine biomarkers, and genetic analyses. Sample size ranged between 308 and 1,574,749 in studies providing prediction approaches for CKD. Validation studies have been found for 15 of the 19 CKD prediction approaches. Identified risk approaches often target T2DM patients. Examples of the different approaches are provided in Table 2 and an overview of occurrences of different prediction parameters used in the identified tools of Table 2 is demonstrated in a word cloud (Fig 4).

In the following, the prediction categories and exemplary approaches identified for CKD are presented. One approach for CKD risk prediction is the use of surveys. Surveys are a simple method using nonlaboratory clinical variables that are routinely available and minimally intrusive. Among others, these surveys include typical risk factors for CKD such as age, hypertension, diabetes mellitus (DM), sex, BMI, anemia, and history of heart diseases. Some of the surveys are self-assessment scores such as QKidney score [104] or the Screening for Occult Renal Disease Score (SCORED) score [106], while others are assessed by physicians [105,107]. Usually, these surveys are based on a scoring system to assess the probability of developing CKD. Some scores provide risk prediction over a certain period of time. For example, the QKidney score evaluates the risk of developing CKD over the next five years [104], while the clinical model of Chien et al. estimates the 4-year risk of incidence CKD [107]. There are tools that cover more than one CRM disease as these diseases share common risk factors. For example, the Rotterdam-Hoorn score [105] was developed for the prediction of CKD, T2DM and CVD.

Another approach to predict the risk of CKD onset is the combination of nonlaboratory clinical factors with biological parameters such as blood biomarkers. Therefore, this approach usually requires the involvement of a physician. While some tests predict the risk of disease onset in terms of a scoring system [97,108], others give a web-based statistical evaluation as a prediction result, such as the Dynamic DN incidence nomogram [100], or a test report such as PromarkerD [103]. Usually, these approaches combine a variety of laboratory parameters, e.g., eGFR, blood glucose, and fatty acids. Some approaches specifically focus on blood glucose measurements [97,115]. For example, Xin et al. constructed a risk score based on hemoglobin glycation index (HGI), which is calculated from the values of HbA1c and fasting plasma glucose (FPG) [97].

Besides approaches that use biological biomarkers found in the blood, other approaches were identified that include biological parameters from urinalysis to predict the risk of CKD onset. Usually, this includes the measurement of protein-based parameters such as proteinuria. While some approaches were identified that combine urinalysis with nonlaboratory clinical parameters, e.g., the Korean Risk Score [109] or the urine dipstick point-of-care model by Bradshaw et al. [110], others focus on urinalysis alone. For example, the DNlite-IVD103 [99] is a urinary enzyme-linked immunoassay (ELISA) test that detects a post translational modified fragment of fetuin-A to predict short-term renal function change in T2DM with microalbuminuria. These approaches were developed to be used at population level by electronically checking for the presence of the variables in patients’ data or as a checklist to estimate individual’s risk of CKD onset.

Some approaches were identified that include clinical predictors as well as biological predictors both from blood and urine. Usually, these factors are combined into a risk score which refers to the probability of CKD occurrence. Some risk scores predict the disease onset over a certain time period. For example, the risk prediction score based on the Framingham Heart Study cohort estimates an individual’s probability of developing CKD over a 10-year period [112] while the biochemical point-based model by Chien et al. predicts CKD over a period of four years [107]. Some risk scores focus on the general population such as the renal risk score based on the Prevention of Renal and Vascular End-stage Disease (PREVEND) study [111], while others focus on T2DM patients, e.g., the quality-of-care scoring system [101]. Mostly, approaches based on clinical and biological predictors from both blood and urine aim to be assessed by physicians to estimate an individual’s risk of developing CKD. The results can be used to support clinical counseling and decision-making at a primary care level.

In addition to traditional clinical and biological CKD risk factors, such as age, hypertension, DM, and blood glucose, there is evidence of genetics influencing the risk of developing CKD. Blech et al. created the multifactorial genetic model which includes genetic components by combining single nucleotide polymorphisms (SNPs) with nonlaboratory clinical predictors of nephropathy as an approach for the prediction of CKD onset [113]. Compared to a similarly constructed non-genetic model, the multifactorial genetic model predicted CKD onset more effectively [113]. Liao et al. created a score to identify T2DM patients at risk of developing DN that combines clinical and blood predictors with 7 SNPs, which were identified in a prior genome-wide association study (GWAS) [114,116]. Compared to traditional factors, their genetic risk score obtains better prediction outcomes. Ma et al. developed risk score which is solely based on genetic loci to predict incident cases of stage 3 CKD [102].

## Discussion

Aim of this scoping review was to provide an overview of different types of approaches for the prediction of T2DM and CKD and their intended use, to inform the reader about the multiple approaches to prediction. Our review identified several different approaches, including methods based on clinical parameters, biological parameters (blood, urine, microbiome, genetics), combinations of both, sequential approaches, and approaches using exposure and lifestyle factors. The identified methods use self-reported and proxy questionnaires, scores with point systems, blood tests, urinalysis, genetic tests, machine learning, and smartphone apps.

Risk surveys, such as the FINDRISC to predict T2DM, have been developed to enable an inexpensive and easily applicable risk prediction at a low threshold. Often, these surveys include simple parameters which are ordinarily available in routine clinical setting. These scores offer potential for risk stratification of entire populations by applying them to computerized medical records to systematically identify those patients who need further investigation or regular monitoring [104].

For CKD, approaches based on urinalysis alone or in combination with clinical predictors have been found to identify people at high-risk of future disease onset. Urinalysis is described as an inexpensive, safe, and non-invasive method which can be easily applied by health care providers. However, O’Seaghdha et al. found that the power of their CKD risk score is predominantly driven by clinical risk factors such as age, T2DM, and hypertension [112]. This is in line with Chien et al. who constructed two models: one based on clinical predictors (clinical model), and one based on clinical and biological predictors from both urine and blood (biochemical model) (see Table 2) [107]. Both models had a similar performance in terms of CKD prediction, indicating that the clinical model is more feasible for the use in primary care [107]. Further research is required in order to adequately assess the cost-effectiveness of the different approaches including clinical predictors, biological predictors and a combination of both.

One systematic review [49] investigated polygenic risk scores for prediction of T2DM and included a number of longitudinal studies. The authors state that the addition of polygenic risk scores to clinical risk factors has failed to show convincing improvements, thus questioning their clinical relevance [49]. However, recent studies have shown promising results regarding the potential to identify individuals with increased genetic risk of developing T2DM [77]. Interestingly, according to a longitudinal study by Balkau et al., the addition of genetic polymorphism to obesity and baseline glucose only contributed little to disease prediction [72]. Among the identified studies that included CKD associated genetic loci in CKD risk prediction, common clinical risk factors alone or in combination with genetic markers seemed to be more useful in disease prediction rather than genetic markers alone [102].

Studies show strong associations between T2DM, deterioration of renal function and the retinal structure [117,118]. In a systematic review, Peng et al. analyzed the detection of systemic diseases from ocular images using artificial intelligence, indicating that scores based on ocular images may provide early insight into systemic diseases prognosis such as CKD [119]. For CKD detection, retinal image-based models showed comparable to slightly better results to models based on traditional risk factors such as age, gender, ethnicity, T2DM, and hypertension [120,121,122]. Positive results from the use of smartphone camera-captured images indicate high real-life feasibility and utility in CKD diagnosis administered via personal mobile devices [120]. Up to this point, ocular image-based approaches are used to detect early stages of CKD and disease progression. Comparable approaches were also found to detect T2DM through retinal images using deep learning [123]. However, as models are already able to predict important risk factors (age, gender, smoking, BMI, dyslipidemia with elevated triglycerides levels, etc.) [124], it might be only a matter of time until ocular-image-based approaches can also be assessed to predict the risk of T2DM and CKD onset.

In recent studies, machine learning is increasingly being used. The use of a machine learning approach is seen to have a revolutionizing potential for T2DM risk prediction and is increasingly being evaluated in the literature [125]. Various techniques are being used, although underlying populations, untidy datasets, and validation issues make generalization difficult, and more transparent reporting would be desirable in many studies [25,45].

Overall, hardly any tools were identified that predict more than one CRM disease. Such approaches would address one of the major challenges in primary care, namely screening for the risk of multiple relevant diseases in a time- and resource-limited setting [126]. The three cardiometabolic diseases share many risk factors and common prevention opportunities have been acknowledged [127]. A joint approach for the identification of those at high risk of developing one of these cardiometabolic diseases may be more effective because it highlights the importance of multiple risk factors [105]. The Rotterdam-Hoorn score was developed as a risk stratification tool to identify people who are at risk for T2DM, CKD and CVD [105], has been validated [128], and has been incorporated into the Dutch guideline for the prevention of cardiometabolic diseases [129]. According to the authors, the Rotterdam Hoorn score can be used in prevention programs to identify persons with an increased risk for CRM diseases who need multifactorial risk assessment and intervention [128].

## Limitations

This review has a number of limitations. First, aim of this scoping review was to capture different types of approaches of prediction, without aspiring to cover all existing approaches of a particular kind. Thus, the approaches presented in the “Results” section are exemplary for the respective category, independent of the accuracy, validity and clinical utility. Also, patient acceptability, frequency of usage, impact on healthcare decision-making, and cost-effectiveness were not considered in this review. Second, included studies were of considerable heterogeneity pertaining to study populations, predictors used, outcome definitions, and statistical methods. However, the method of scoping review primarily aims to collate the available literature and identify key concepts and thus, heterogeneity was not considered. Third, as most of the identified approaches were derived from Caucasian and Asian populations, it is not clear if they are transferable to other populations, given the different biological factors involved in the development of T2DM and CKD. Fourth, the literature searches were carried out in a stepwise manner. While this approach was considered to be most reasonable and practical for fulfilling the aim of the scoping review, it cannot be ruled out that approaches for which no reviews were available in the first step were missed. Also, the adaptation of search strategies to the different databases may have led to different search results. On the other hand, the combination of these three bibliographic databases and Google Scholar has shown to achieve a broad and efficient coverage [130].

## Conclusion

T2DM and CKD have a strong societal and economic impact and often remain undiagnosed for a long period. Identifying persons at risk is essential for prevention and early action. Therefore, an overview of different tools for the risk prediction of type 2 diabetes mellitus or chronic kidney disease is provided in this scoping review. As cardio-renal-metabolic diseases share predicting factors, approaches that address multiple relevant diseases such as type 2 diabetes mellitus, chronic kidney disease and cardiovascular disease can be of great interest, especially in time- and resource-constrained healthcare settings.

## Supporting information

S1 Appendix Full search strategies and number of hits per keyword for T2DM. (DOCX)

S2 Appendix Full search strategies and number of hits per keyword for CKD. (DOCX)

S3 Appendix Systematic reviews for T2DM that have been identified in step one and did not meet the inclusion criteria but indicate further approaches that have not been covered in step one. (DOCX)

S4 Appendix Study characteristics of identified literature about prediction approaches for T2DM. (DOCX)

S5 Appendix Systematic reviews for CKD that have been identified in step one and did not meet the inclusion criteria but indicate further approaches that have not been covered in step one. (DOCX)

S6 Appendix Study characteristics of identified literature about prediction approaches for CKD. (DOCX)

S7 Appendix Additional information on the identified approaches. (DOCX)

## Abbreviations

AAA: aromatic amino acids
AA: amino acids
ADA: American Diabetes Association
ADVANCE: Action in Diabetes and Vascular Disease: Preterax and Diamicron MR Controlled Evaluation
AI: Artificial Intelligence
ApoA4: apolipoprotein A-IV
ARIC: study, Atherosclerosis Risk in Communities study
AUSDRISK: Australian Type 2 Diabetes Risk Assessment Tool
BCAA: branched chained amino acids
BMI: body mass index
BP: blood pressure
CANRISK: Canadian Diabetes Risk Questionnaire
CD5L: CD5 antigen-like
CGM: continuous glucose monitoring
CHS: cardiovascular health study
CKD: chronic kidney disease
CRM: cardio-renal-metabolic
CRP: C-reactive protein
CVD: cardiovascular disease
DBP: diastolic blood pressure
DESIR: Data from the Epidemiological Study on the Insulin Resistance Syndrome
DKD: diabetic kidney disease
DL: deep learning
DM: diabetes mellitus
DN: diabetic nephropathy
DNA: deoxyribonucleic acid
DNAm: DNA methylation
DPB: diastolic blood pressure
DPoRT: Diabetes Population Risk Tool
ELISA: enzyme-linked immunoassay test
EMBASE: Excerpta Medica Database
eGFR: estimated glomerular filtration rate
FINDRISC: Finnish Diabetes Risk Score
FPG: fasting plasma glucose
GWAS: genome-wide association study
HbA1c: glycated hemoglobin
HbA1c-CV: glycated hemoglobin coefficient of variation
HDL: high-density lipoprotein
HF: heart failure
HGI: hemoglobin glycation index
IBS: Intelligent Bio Solutions
IDRS: Indian Diabetes risk score
IGFBP3: insulin-like growth factor-binding protein 3
JBI: Joanna Briggs Institute
LDL: low-density-lipoprotein
Medline: Medical Literature Analysis and Retrieval System Online
NHANES: Diabetes Risk Calculator based on the Third National Health and Nutrition Examination Survey
OGTT: oral glucose tolerance test
OSF: Open Science Framework
PRISMA: Preferred Reporting Items for Systematic Reviews and Meta-Analyses
PREVEND: Prevention of Renal and Vascular End-stage Disease
PVD: Peripheral vascular disease
RF: random forest
SBP: systolic blood pressure
SCORED: Screening for Occult Renal Disease
sKlotho: soluble or secreted Klotho
SNPs: single nucleotide polymorphisms
T2DM: type II diabetes mellitus
TIA: transient ischemic attack
TOI: transdermal optical imaging
UAE: urinary albumin excretion
UKB: UK Biobank
VAI: Visceral Adiposity Index
