# Supplementary material for: Approaches to predict future type 2 diabetes mellitus and chronic kidney disease: A scoping review
Source: PLoS One. 2025 Jun 11;20(6):e0325182. doi: 10.1371/journal.pone.0325182 (PMC12157063; doi:10.1371/journal.pone.0325182)
Supplement: S1 Appendix — (DOCX) [file pone.0325182.s001.docx]

**S1 Appendix. Full search strategies and number of hits per keyword for T2DM**

# Search for systematic reviews

## **PubMed**

(predict*[Title/Abstract] OR risk[MESH] OR risk*[Title/Abstract] OR prognos*[Title/Abstract] OR indic*[Title/Abstract]) AND (approach*[Title/Abstract] OR assessment[Title/Abstract] OR score*[Title/Abstract] OR instrument*[Title/Abstract] OR tool*[Title/Abstract] OR model*[Title/Abstract]) AND (type II diabetes[Title/Abstract] OR type 2 diabetes[Title/Abstract] OR diabetes mellitus, type 2 [MESH] OR T2D[Title/Abstract] OR T2DM[Title/Abstract])

| Platform | PubMed | |
| --- | --- | --- |
| Filters | Study type: systematic reviews | |
|  |  |  |
| 1a | risk*[Title/Abstract] | 102,248 |
| 1b | indic*[Title/Abstract] | 39,086 |
| 1c | risk[MESH] | 24,749 |
| 1d | predict*[Title/Abstract] | 18,661 |
| 1e | prognos*[Title/Abstract] | 12,889 |
| 1f | 1a OR 1b OR 1c OR 1d OR 1e | 140,790 |
|  |  |  |
| 2a | model*[Title/Abstract] | 47,960 |
| 2b | assessment[Title/Abstract] | 42,751 |
| 2c | approach*[Title/Abstract] | 31,336 |
| 2d | tool*[Title/Abstract] | 28,932 |
| 2e | score*[Title/Abstract] | 24,618 |
| 2f | instrument*[Title/Abstract] | 7,884 |
| 2g | 2a OR 2b OR 2c OR 2d OR 2e OR 2f | 128,142 |
|  |  |  |
| 3a | type 2 diabetes[Title/Abstract] | 4,192 |
| 3b | diabetes mellitus, type 2 [MESH] | 3,622 |
| 3c | T2DM[Title/Abstract] | 1,238 |
| 3d | T2D[Title/Abstract] | 416 |
| 3e | type II diabetes[Title/Abstract] | 112 |
| 3f | 3a OR 3b OR 3c OR 3d OR 3e OR 3e | 4,911 |
|  |  |  |
| 4a | 1f AND 2g AND 3f | 1,762 |

## **EMBASE**

('predict*':ab,ti OR 'risk'/exp/mj OR 'risk*':ab,ti OR 'prognos*':ab,ti OR 'indic*':ab,ti) AND ('approach':ab,ti OR 'assessment':ab,ti OR 'scor*':ab,ti OR 'instrument*':ab,ti OR 'tool*':ab,ti OR 'model':ab,ti) AND ('non insulin dependent diabetes mellitus'/exp OR 'non insulin dependent diabetes mellitus':ab,ti OR 'type II diabetes':ab,ti OR 'T2D':ab,ti OR 'T2DM':ab,ti) AND 'systematic review'/de

| Platform | EMBASE | |
| --- | --- | --- |
| Filters | Study type: systematic reviews | |
|  |  |  |
| 1a | 'risk*':ab,ti | 145,995 |
| 1b | 'indic*':ab,ti | 57,120 |
| 1c | 'predict*':ab,ti | 30,452 |
| 1d | 'prognos*':ab,ti | 21,979 |
| 1e | 'risk'/exp/mj | 11,943 |
| 1f | 1a OR 1b OR 1c OR 1d OR 1e | 202,135 |
|  |  |  |
| 2a | 'assessment':ab,ti | 55,750 |
| 2b | 'model':ab,ti | 49,026 |
| 2c | 'tool*':ab,ti | 39,681 |
| 2d | 'scor*':ab,ti | 35,557 |
| 2e | 'approach':ab,ti | 35,540 |
| 2f | 'instrument*':ab,ti | 10,157 |
| 2g | 2a OR 2b OR 2c OR 2d OR 2e OR 2f | 162,381 |
|  |  |  |
| 3a | 'non insulin dependent diabetes mellitus'/exp | 8,982 |
| 3b | 'T2DM':ab,ti | 2,186 |
| 3c | 'T2D':ab,ti | 736 |
| 3d | 'type II diabetes':ab,ti | 178 |
| 3e | 'non insulin dependent diabetes mellitus':ab,ti | 11 |
| 3f | 3a OR 3b OR 3c OR 3d OR 3e | 9,218 |
|  |  |  |
| 4a | 1f AND 2g AND 3f | 2,400 |

## **Web of Science**

(TI=(risk*) OR TI=(predict*) OR TI=(prognos*) OR TI=(indic*) OR AB=(risk*) OR AB=(predict*) OR AB=(prognos*) OR AB=(indic*)) AND (TI=(approach) OR TI=(assessment) OR TI=(scor*) OR TI=(instrument*) OR TI=(tool*) OR TI=(model) OR AB=(approach) OR AB=(assessment) OR AB=(scor*) OR AB=(instrument*) OR AB=(tool*) OR AB=(model)) AND (TI=(type 2 diabetes mellitus) OR TI=(type II diabetes) OR TI=(T2D) OR TI=(T2DM) OR TI=(non insulin dependent diabetes mellitus) OR AB=(type 2 diabetes mellitus) OR AB=(type II diabetes) OR AB=(T2D) OR AB=(T2DM) OR AB=(non insulin dependent diabetes mellitus)) AND (TI=(systematic review) OR AB=(systematic review))

| Platform | Web of Science | |
| --- | --- | --- |
| Filters | systematic reviews | |
|  |  |  |
| 1a | TI=(risk*) OR AB=(risk*) | 107,451 |
| 1b | TI=(indic*) OR AB=(indic*) | 47,623 |
| 1c | TI=(predict*) OR AB=(predict*) | 24,308 |
| 1d | TI=(prognos*) OR AB=(prognos*) | 14,205 |
| 1e | 1a OR 1b OR 1c OR 1d | 164,474 |
|  |  |  |
| 2a | TI=(model) OR AB=(model) | 59,806 |
| 2b | TI=(assessment) OR AB=(assessment) | 54,816 |
| 2c | TI=(approach) OR AB=(approach) | 54,508 |
| 2d | TI=(tool*) OR AB=(tool*) | 38,830 |
| 2e | TI=(scor*) OR AB=(scor*) | 26,033 |
| 2f | TI=(instrument*) OR AB=(instrument*) | 10,231 |
| 2g | 2a OR 2b OR 2c OR 2d OR 2e OR 2f | 179,498 |
|  |  |  |
| 3a | (TI=(type 2 diabetes mellitus) OR AB=(type 2 diabetes mellitus) | 2,698 |
| 3b | TI=(T2DM) OR AB=(T2DM) | 1,328 |
| 3c | TI=(T2D) OR AB=(T2D) | 420 |
| 3d | TI=(type II diabetes) OR AB=(type II diabetes) | 269 |
| 3e | TI=(non insulin dependent diabetes mellitus) OR AB=(non insulin dependent diabetes mellitus) | 18 |
| 3f | 3a OR 3b OR 3c OR 3d OR 3e | 3,626 |
|  |  |  |
| 4a | 1e AND 2g AND 3f | 1,178 |

# Search for primary literature

## **PubMed**

(predict*[Title/Abstract] OR risk[MESH] OR risk*[Title/Abstract] OR prognos*[Title/Abstract] OR indic*[Title/Abstract]) AND (approach*[Title/Abstract] OR assessment[Title/Abstract] OR score*[Title/Abstract] OR instrument*[Title/Abstract] OR tool*[Title/Abstract] OR model*[Title/Abstract]) AND (type II diabetes[Title/Abstract] OR type 2 diabetes[Title/Abstract] OR diabetes mellitus, type 2[MESH] OR T2D[Title/Abstract] OR T2DM[Title/Abstract]) AND (branched-chain amino acid[Title/Abstract] OR BCAA[Title/Abstract] OR branched-chain amino acid[Mesh] OR fetuin-A[Mesh] OR fetuin-A[Title/Abstract] OR Gastrointestinal Microbiome[Mesh] OR Microbiota[Mesh] OR microbiome*[Title/Abstract] OR microflora*[Title/Abstract] OR microbiota*[Title/Abstract] OR microbial[Title/Abstract] OR continuous glucose monitor*[Title/Abstract] OR Dexcom[Title/Abstract] OR Abbott[Title/Abstract] OR Medtronic[Title/Abstract] OR Senseonics[Title/Abstract])

| Platform | PubMed | |
| --- | --- | --- |
|  |  |  |
| 1a | indic*[Title/Abstract] | 3,956,664 |
| 1b | risk*[Title/Abstract] | 2,862,475 |
| 1c | predict*[Title/Abstract] | 2,017,934 |
| 1d | risk[MESH] | 1,375,064 |
| 1e | prognos*[Title/Abstract] | 792,254 |
| 1f | 1a OR 1b OR 1c OR 1d OR 1e | 8,397,951 |
|  |  |  |
| 2a | model*[Title/Abstract] | 3,711,325 |
| 2b | approach*[Title/Abstract] | 2,298,937 |
| 2c | assessment[Title/Abstract] | 1,238,227 |
| 2d | score*[Title/Abstract] | 1,234,835 |
| 2e | tool*[Title/Abstract] | 963,085 |
| 2f | instrument*[Title/Abstract] | 347,521 |
| 2g | 2a OR 2b OR 2c OR 2d OR 2e OR 2f | 7,855,883 |
|  |  |  |
| 3a | diabetes mellitus, type 2[MESH] | 168,838 |
| 3b | type 2 diabetes[Title/Abstract] | 164,505 |
| 3c | T2D[Title/Abstract] | 16,134 |
| 3d | T2DM[Title/Abstract] | 31,74 |
| 3e | type II diabetes[Title/Abstract] | 9,896 |
| 3f | 3a OR 3b OR 3c OR 3d OR 3e | 231,605 |
|  |  |  |
| 4a | microbial[Title/Abstract] | 238,133 |
| 4b | microbiota*[Title/Abstract] | 84,106 |
| 4c | Microbiota[Mesh] | 72,883 |
| 4d | microbiome*[Title/Abstract] | 55,207 |
| 4e | branched-chain amino acid[Mesh] | 54,623 |
| 4f | Gastrointestinal Microbiome[Mesh] | 37,113 |
| 4g | microflora*[Title/Abstract] | 18,407 |
| 4h | Abbott[Title/Abstract] | 8,720 |
| 4i | continuous glucose monitor*[Title/Abstract] | 6,794 |
| 4j | Medtronic[Title/Abstract] | 6,291 |
| 4k | branched-chain amino acid[Title/Abstract] | 3,062 |
| 4l | BCAA[Title/Abstract] | 2,827 |
| 4m | fetuin-A[Title/Abstract] | 1,428 |
| 4n | fetuin-A[Mesh] | 1,122 |
| 4o | Dexcom[Title/Abstract] | 324 |
| 4p | Senseonics[Title/Abstract] | 13 |
| 4q | 4a OR 4b OR 4c OR 4d OR 4e OR 4f OR 4g… OR 4p | 411,643 |
|  |  |  |
| 5a | 1f AND 2g AND 3f AND 4q | 1,053 |
|  |  |  |
| 6a | filter from 2016 | 845 |

## **EMBASE**

('predict*':ab,ti OR 'risk'/exp/mj OR 'risk*':ab,ti OR 'prognos*':ab,ti OR 'indic*':ab,ti) AND ('approach':ab,ti OR 'assessment':ab,ti OR 'scor*':ab,ti OR 'instrument*':ab,ti OR 'tool*':ab,ti OR 'model':ab,ti) AND ('non insulin dependent diabetes mellitus'/exp OR 'non insulin dependent diabetes mellitus':ab,ti OR 'type II diabetes':ab,ti OR 'T2D':ab,ti OR 'T2DM':ab,ti) AND ('branched chain amino acid':ab,ti OR bcaa:ab,ti OR 'fetuin a':ab,ti OR 'microbiome'/exp/mj OR 'microbiome analysis':ab,ti OR microbiomics:ab,ti OR microbiome:ab,ti OR microflora:ab,ti OR microbial:ab,ti OR 'continuous glucose monitoring system':ab,ti OR Dexcom:ab,ti OR Abbott:ab,ti OR Medtronic:ab,ti OR Senseonics:ab,ti)

| Platform | EMBASE | |
| --- | --- | --- |
|  |  |  |
| 1a | indic*':ab,ti | 4,931,418 |
| 1b | risk*':ab,ti | 4,096,525 |
| 1c | predict*':ab,ti | 2,695,633 |
| 1d | prognos*':ab,ti | 1,171,242 |
| 1e | risk'/exp/mj | 411,212 |
| 1f | 1a OR 1b OR 1c OR 1d OR 1e | 10,588,585 |
|  |  |  |
| 2a | model':ab,ti | 3,326,345 |
| 2b | approach':ab,ti | 2,133,360 |
| 2c | scor*':ab,ti | 1,993,939 |
| 2d | assessment':ab,ti | 1,734,787 |
| 2e | tool*':ab,ti | 1,274,811 |
| 2f | instrument*':ab,ti | 439,729 |
| 2g | 2a OR 2b OR 2c OR 2d OR 2e OR 2f | 8,802,289 |
|  |  |  |
| 3a | non insulin dependent diabetes mellitus'/exp | 328,762 |
| 3b | T2DM':ab,ti | 52,415 |
| 3c | T2D':ab,ti | 29,108 |
| 3d | type II diabetes':ab,ti | 15,57 |
| 3e | non insulin dependent diabetes mellitus':ab,ti | 7,853 |
| 3f | 3a OR 3b OR 3c OR 3d OR 3e | 345,83 |
|  |  |  |
| 4a | microbial:ab,ti | 263,643 |
| 4b | microbiome:ab,ti | 58,469 |
| 4c | microflora:ab,ti | 21,276 |
| 4d | Abbott:ab,ti | 17,891 |
| 4e | microbiome'/exp/mj | 16,076 |
| 4f | Medtronic:ab,ti | 14,709 |
| 4g | bcaa:ab,ti | 3,637 |
| 4h | branched chain amino acid':ab,ti | 3,538 |
| 4i | fetuin a':ab,ti | 2,127 |
| 4j | microbiome analysis':ab,ti | 1,857 |
| 4k | continuous glucose monitoring system':ab,ti | 1,542 |
| 4l | Dexcom:ab,ti | 1,140 |
| 4m | microbiomics:ab,ti | 268 |
| 4n | Senseonics:ab,ti | 40 |
| 4o | 4a OR 4b OR 4c OR 4d OR 4e OR 4f OR 4g… OR 4n | 358,111 |
|  |  |  |
| 5a | 1f AND 2g AND 3f AND 4o | 603 |
|  |  |  |
| 6a | filter from 2016 | 469 |

## **Web of Science**

(TI=(risk*) OR TI=(predict*) OR TI=(prognos*) OR TI=(indic*) OR AB=(risk*) OR AB=(predict*) OR AB=(prognos*) OR AB=(indic*)) AND (TI=(approach) OR TI=(assessment) OR TI=(scor*) OR TI=(instrument*) OR TI=(tool*) OR TI=(model) OR AB=(approach) OR AB=(assessment) OR AB=(scor*) OR AB=(instrument*) OR AB=(tool*) OR AB=(model)) AND (TI=(type 2 diabetes mellitus) OR TI=(type II diabetes) OR TI=(T2D) OR TI=(T2DM) OR TI=(non insulin dependent diabetes mellitus) OR AB=(type 2 diabetes mellitus) OR AB=(type II diabetes) OR AB=(T2D) OR AB=(T2DM) OR AB=(non insulin dependent diabetes mellitus)) AND (TI=(branched chain amino acid) OR AB=(branched chain amino acid) OR TI=(bcaa) OR AB=(bcaa) OR TI=(fetuin a) OR AB=(fetuin a) OR TI=(microbiom*) OR AB=(microbiom*) OR TI=(microflora) OR AB=(microflora) OR TI=(microbial) OR AB=(microbial) OR TI=(continuous glucose monitoring system) OR AB=(continuous glucose monitoring system) OR TI=(dexcom) OR AB=(dexcom) OR TI=(abbott) OR AB=(abbott) OR TI=(medtronic) OR AB=(medtronic) OR TI=(Senseonics) OR AB=(Senseonics))

| Platform | Web of Science | |
| --- | --- | --- |
|  |  |  |
| 1a | TI=(indic*) OR AB=(indic*) | 5,867,426 |
| 1b | TI=(predict*) OR AB=(predict*) | 3,557,073 |
| 1c | TI=(risk*) OR AB=(risk*) | 3,165,471 |
| 1d | TI=(prognos*) OR AB=(prognos*) | 734,335 |
| 1e | 1a OR 1b OR 1c OR 1d | 11,509,358 |
|  |  |  |
| 2a | TI=(model) OR AB=(model) | 8,462,025 |
| 2b | TI=(approach) OR AB=(approach) | 4,640,698 |
| 2c | TI=(assessment) OR AB=(assessment) | 1,819,842 |
| 2d | TI=(tool*) OR AB=(tool*) | 1,625,260 |
| 2e | TI=(scor*) OR AB=(scor*) | 1,384,770 |
| 2f | TI=(instrument*) OR AB=(instrument*) | 612,652 |
| 2g | 2a OR 2b OR 2c OR 2d OR 2e OR 2f | 14,947,590 |
|  |  |  |
| 3a | TI=(type 2 diabetes mellitus) OR AB=(type 2 diabetes mellitus) | 80,609 |
| 3b | TI=(T2DM) OR AB=(T2DM) | 31,718 |
| 3c | TI=(type II diabetes) OR AB=(type II diabetes) | 20,139 |
| 3d | TI=(T2D) OR AB=(T2D) | 16,235 |
| 3e | TI=(non insulin dependent diabetes mellitus) OR AB=(non insulin dependent diabetes mellitus) | 7,757 |
| 3f | 3a OR 3b OR 3c OR 3d OR 3e | 123,027 |
|  |  |  |
| 4a | TI=(microbial) OR AB=(microbial) | 343,052 |
| 4b | TI=(microbiom*) OR AB=(microbiom*) | 55,716 |
| 4c | TI=(microflora) OR AB=(microflora) | 20,860' |
| 4d | TI=(branched chain amino acid) OR AB=(branched chain amino acid) | 9,538 |
| 4e | TI=(abbott) OR AB=(abbott) | 9,452 |
| 4f | TI=(medtronic) OR AB=(medtronic) | 5,438 |
| 4g | TI=(continuous glucose monitoring system) OR AB=(continuous glucose monitoring system) | 2,866 |
| 4h | TI=(fetuin a) OR AB=(fetuin a) | 2,810' |
| 4i | TI=(bcaa) OR AB=(bcaa) | 2,601 |
| 4j | TI=(dexcom) OR AB=(dexcom) | 289 |
| 4k | TI=(Senseonics) OR AB=(Senseonics) | 9 |
| 4l | 4a OR 4b OR 4c OR 4d OR 4e OR 4f OR 4g… OR 4k | 422,208 |
|  |  |  |
| 5a | 1e AND 2g AND 3f AND 4l | 325 |
|  |  |  |
| 6a | filter from 2016 | 279 |
