# Supplementary material for: Approaches to predict future type 2 diabetes mellitus and chronic kidney disease: A scoping review
Source: PLoS One. 2025 Jun 11;20(6):e0325182. doi: 10.1371/journal.pone.0325182 (PMC12157063; doi:10.1371/journal.pone.0325182)
Supplement: S2 Appendix — (DOCX) [file pone.0325182.s002.docx]

**S2 Appendix. Full search strategies and number of hits per keyword for CKD**

# Search for systematic reviews

## **PubMed**

(predict*[Title/Abstract] OR risk[MESH] OR risk*[Title/Abstract] OR prognos*[Title/Abstract] OR indic*[Title/Abstract]) AND (approach*[Title/Abstract] OR assessment[Title/Abstract] OR score*[Title/Abstract] OR instrument*[Title/Abstract] OR tool*[Title/Abstract] OR model*[Title/Abstract]) AND (chronic kidney disease[Title/Abstract] OR CKD[Title/Abstract] OR chronic renal disease[Title/Abstract] OR diabetic kidney disease[Title/Abstract] OR DKD[Title/Abstract])

| Platform | PubMed | |
| --- | --- | --- |
| Filters | Study type: systematic reviews | |
|  |  |  |
| 1a | risk*[Title/Abstract] | 102,248 |
| 1b | indic*[Title/Abstract] | 39,086 |
| 1c | risk[MESH] | 24,749 |
| 1d | predict*[Title/Abstract] | 18,661 |
| 1e | prognos*[Title/Abstract] | 12,889 |
| 1f | 1a OR 1b OR 1c OR 1d OR 1e | 140,790 |
|  |  |  |
| 2a | model*[Title/Abstract] | 47,960 |
| 2b | assessment[Title/Abstract] | 42,751 |
| 2c | approach*[Title/Abstract] | 31,336 |
| 2d | tool*[Title/Abstract] | 28,932 |
| 2e | score*[Title/Abstract] | 24,618 |
| 2f | instrument*[Title/Abstract] | 7,884 |
| 2g | 2a OR 2b OR 2c OR 2d OR 2e OR 2f | 128,142 |
|  |  |  |
| 3a | chronic kidney disease[Title/Abstract] | 1,683 |
| 3b | CKD[Title/Abstract] | 1,155 |
| 3c | diabetic kidney disease[Title/Abstract] | 99 |
| 3d | DKD[Title/Abstract] | 63 |
| 3e | chronic renal disease[Title/Abstract] | 32 |
| 3f | 3a OR 3b OR 3c OR 3d OR 3e | 1,853 |
|  |  |  |
| 4a | 1f AND 2g AND 3f | 707 |

## **EMBASE**

('predict*':ab,ti OR 'risk'/exp/mj OR 'risk*':ab,ti OR 'prognos*':ab,ti OR 'indic*':ab,ti) AND ('approach':ab,ti OR 'assessment':ab,ti OR 'scor*':ab,ti OR 'instrument*':ab,ti OR 'tool*':ab,ti OR 'model':ab,ti) AND ('chronic kidney failure'/exp OR 'chronic renal disease':ab,ti OR 'CKD':ab,ti OR 'chronic kidney disease':ab,ti OR 'diabetic kidney disease':ab,ti OR DKD:ab,ti) AND 'systematic review'/de

| Platform | EMBASE | |
| --- | --- | --- |
| Filters | Study type: systematic reviews | |
|  |  |  |
| 1a | 'risk*':ab,ti | 145,995 |
| 1b | 'indic*':ab,ti | 57,120 |
| 1c | 'predict*':ab,ti | 30,452 |
| 1d | 'prognos*':ab,ti | 21,979 |
| 1e | 'risk'/exp/mj | 11,943 |
| 1f | 1a OR 1b OR 1c OR 1d OR 1e | 202,135 |
|  |  |  |
| 2a | 'assessment':ab,ti | 55,750 |
| 2b | 'model':ab,ti | 49,026 |
| 2c | 'tool*':ab,ti | 39,681 |
| 2d | 'scor*':ab,ti | 35,557 |
| 2e | 'approach':ab,ti | 35,540 |
| 2f | 'instrument*':ab,ti | 10,157 |
| 2g | 2a OR 2b OR 2c OR 2d OR 2e OR 2f | 162,381 |
|  |  |  |
| 3a | 'chronic kidney failure'/exp | 3,796 |
| 3b | 'chronic kidney disease':ab,ti | 2,819 |
| 3c | 'CKD':ab,ti | 1,953 |
| 3d | 'diabetic kidney disease':ab,ti | 166 |
| 3e | 'DKD':ab,ti | 107 |
| 3f | 'chronic renal disease':ab,ti | 70 |
| 3g | 3a OR 3b OR 3c OR 3d OR 3e OR 3f | 4,369 |
|  |  |  |
| 4a | 1f AND 2g AND 3g | 1,191 |

## **Web of Science**

(TI=(risk*) OR TI=(predict*) OR TI=(prognos*) OR TI=(indic*) OR AB=(risk*) OR AB=(predict*) OR AB=(prognos*) OR AB=(indic*)) AND (TI=(approach) OR TI=(assessment) OR TI=(scor*) OR TI=(instrument*) OR TI=(tool*) OR TI=(model) OR AB=(approach) OR AB=(assessment) OR AB=(scor*) OR AB=(instrument*) OR AB=(tool*) OR AB=(model)) AND (TI=(chronic kidney failure) OR TI=(chronic renal disease) OR TI=(CKD) OR TI=(chronic kidney disease) OR TI=(diabetic kidney disease) OR TI=(DKD) OR AB=(chronic kidney failure) OR AB=(chronic renal disease) OR AB=(CKD) OR AB=(chronic kidney disease) OR AB=(diabetic kidney disease) OR AB=(DKD)) AND (TI=(systematic review) OR AB=(systematic review))

| Platform | Web of Science | |
| --- | --- | --- |
| Filters | systematic reviews | |
|  |  |  |
| 1a | TI=(risk*) OR AB=(risk*) | 107,451 |
| 1b | TI=(indic*) OR AB=(indic*) | 47,623 |
| 1c | TI=(predict*) OR AB=(predict*) | 24,308 |
| 1d | TI=(prognos*) OR AB=(prognos*) | 14,205 |
| 1e | 1a OR 1b OR 1c OR 1d | 164,474 |
|  |  |  |
| 2a | TI=(model) OR AB=(model) | 59,806 |
| 2b | TI=(assessment) OR AB=(assessment) | 54,816 |
| 2c | TI=(approach) OR AB=(approach) | 54,508 |
| 2d | TI=(tool*) OR AB=(tool*) | 38,830 |
| 2e | TI=(scor*) OR AB=(scor*) | 26,033 |
| 2f | TI=(instrument*) OR AB=(instrument*) | 10,231 |
| 2g | 2a OR 2b OR 2c OR 2d OR 2e OR 2f | 179,498 |
|  |  |  |
| 3a | TI=(chronic kidney disease) OR AB=(chronic kidney disease) | 2,018 |
| 3b | TI=(CKD) OR AB=(CKD) | 1144 |
| 3c | TI=(chronic renal disease) OR AB=(chronic renal disease) | 915 |
| 3d | TI=(chronic kidney failure) OR AB=(chronic kidney failure) | 351 |
| 3e | TI=(diabetic kidney disease) OR AB=(diabetic kidney disease) | 230 |
| 3f | TI=(DKD) OR AB=(DKD) | 58 |
| 3g | 3a OR 3b OR 3c OR 3d OR 3e OR 3f | 2,601 |
|  |  |  |
| 4a | 1e AND 2g AND 3g | 817 |

# Search for primary literature

## **PubMed**

(predict*[Title/Abstract] OR risk[MESH] OR risk*[Title/Abstract] OR prognos*[Title/Abstract] OR indic*[Title/Abstract]) AND (approach*[Title/Abstract] OR assessment[Title/Abstract] OR score*[Title/Abstract] OR instrument*[Title/Abstract] OR tool*[Title/Abstract] OR model*[Title/Abstract]) AND (chronic kidney disease[Title/Abstract] OR CKD [Title/Abstract] OR chronic renal disease[Title/Abstract] OR diabetic kidney disease[Title/Abstract] OR DKD[Title/Abstract]) AND (Klotho[Title/Abstract] OR optical coherence tomography[Title/Abstract] OR eye[Title/Abstract] OR retina*[Title/Abstract] OR ocular[Title/Abstract] OR HbA1c[Title/Abstract] OR glycated hemoglobin[Title/Abstract] OR glycated haemoglobin[Title/Abstract] OR glycosylated haemoglobin[Title/Abstract] OR glycosylated hemoglobin[Title/Abstract]) AND (2019/1/15:3000/12/12[pdat])

| Platform | PubMed | |
| --- | --- | --- |
|  |  |  |
| 1a | indic*[Title/Abstract] | 3,956,664 |
| 1b | risk*[Title/Abstract] | 2,862,475 |
| 1c | predict*[Title/Abstract] | 2,017,934 |
| 1d | risk[MESH] | 1,375,064 |
| 1e | prognos*[Title/Abstract] | 792,254 |
| 1f | 1a OR 1b OR 1c OR 1d OR 1e | 8,397,951 |
|  |  |  |
|  |  |  |
| 2a | model*[Title/Abstract] | 3,711,325 |
| 2b | approach*[Title/Abstract] | 2,298,937 |
| 2c | assessment[Title/Abstract] | 1,238,227 |
| 2d | score*[Title/Abstract] | 1,234,835 |
| 2e | tool*[Title/Abstract] | 963,085 |
| 2f | instrument*[Title/Abstract] | 347,521 |
| 2g | 2a OR 2b OR 2c OR 2d OR 2e OR 2f | 7,855,883 |
|  |  |  |
| 3a | chronic kidney disease[Title/Abstract] | 70,781 |
| 3b | CKD [Title/Abstract] | 42,494 |
| 3c | diabetic kidney disease[Title/Abstract] | 4,702 |
| 3d | chronic renal disease[Title/Abstract] | 3,767 |
| 3e | DKD[Title/Abstract] | 2,327 |
| 3f | 3a OR 3b OR 3c OR 3d OR 3e | 82,745 |
|  |  |  |
| 4a | eye[Title/Abstract] | 299,866 |
| 4b | retina*[Title/Abstract] | 223,789 |
| 4c | ocular[Title/Abstract] | 151,142 |
| 4d | HbA1c[Title/Abstract] | 49,735 |
| 4e | optical coherence tomography[Title/Abstract] | 49,658 |
| 4f | glycated hemoglobin[Title/Abstract] | 11,639 |
| 4g | glycosylated hemoglobin[Title/Abstract] | 8,987 |
| 4h | glycated haemoglobin[Title/Abstract] | 4,652 |
| 4i | Klotho[Title/Abstract] | 3,238 |
| 4j | glycosylated haemoglobin[Title/Abstract] | 2,639 |
| 4k | 4a OR 4b OR 4c OR 4d OR 4e OR 4f OR 4g… OR 4j | 639,983 |
|  |  |  |
| 5a | 1f OR 2g OR 3f OR 4k | 670 |
|  |  |  |
| 6a | filter from 15.01.2019 | 383 |

## **EMBASE**

('predict*':ab,ti OR 'risk'/exp/mj OR 'risk*':ab,ti OR 'prognos*':ab,ti OR 'indic*':ab,ti) AND ('approach':ab,ti OR 'assessment':ab,ti OR 'scor*':ab,ti OR 'instrument*':ab,ti OR 'tool*':ab,ti OR 'model':ab,ti) AND ('chronic kidney failure'/exp OR 'chronic renal disease':ab,ti OR 'CKD':ab,ti OR 'chronic kidney disease':ab,ti OR 'diabetic kidney disease':ab,ti OR 'DKD':ab,ti) AND ('Klotho':ab,ti OR 'klotho protein'/exp OR 'optical coherence tomography':ab,ti OR 'eye':ab,ti OR 'retina*':ab,ti OR 'ocular':ab,ti OR 'HbA1c':ab,ti OR 'glycated hemoglobin':ab,ti)

| Platform | EMBASE | |
| --- | --- | --- |
|  |  |  |
| 1a | indic*':ab,ti | 4,931,418 |
| 1b | risk*':ab,ti | 4,096,525 |
| 1c | predict*':ab,ti | 2,695,633 |
| 1d | prognos*':ab,ti | 1,171,242 |
| 1e | risk'/exp/mj | 411,212 |
| 1f | 1a OR 1b OR 1c OR 1d OR 1e | 10,588,585 |
|  |  |  |
| 2a | model':ab,ti | 3,326,345 |
| 2b | approach':ab,ti | 2,133,360 |
| 2c | scor*':ab,ti | 1,993,939 |
| 2d | assessment':ab,ti | 1,734,787 |
| 2e | tool*':ab,ti | 1,274,811 |
| 2f | instrument*':ab,ti | 439,729 |
| 2g | 2a OR 2b OR 2c OR 2d OR 2e OR 2f | 8,802,289 |
|  |  |  |
| 3a | chronic kidney failure'/exp | 199,814 |
| 3b | chronic kidney disease':ab,ti | 110,03 |
| 3c | diabetic kidney disease':ab,ti | 6,535 |
| 3d | CKD':ab,ti | 79,308 |
| 3e | chronic renal disease':ab,ti | 5,635 |
| 3f | DKD':ab,ti | 3,661 |
| 3g | 3a OR 3b OR 3c OR 3d OR 3e OR 3f | 239,247 |
|  |  |  |
| 4a | eye':ab,ti | 366,231 |
| 4b | retina*':ab,ti | 277,867 |
| 4c | ocular':ab,ti | 189,321 |
| 4d | HbA1c':ab,ti | 80,144 |
| 4e | optical coherence tomography':ab,ti | 64,572 |
| 4f | glycated hemoglobin':ab,ti | 15,129 |
| 4g | klotho protein'/exp | 4,365 |
| 4h | klotho protein':ab,ti | 658 |
| 4i | 4a OR 4b OR 4c OR 4d OR 4e OR 4f OR 4g… OR 4h | 799,936 |
|  |  |  |
| 5a | 1f OR 2g OR 3g OR 4i | 1205 |
|  |  |  |
| 6a | filter from 15.01.2019 | 596 |

## **Web of Science**

(TI=(risk*) OR TI=(predict*) OR TI=(prognos*) OR TI=(indic*) OR AB=(risk*) OR AB=(predict*) OR AB=(prognos*) OR AB=(indic*)) AND (TI=(approach) OR TI=(assessment) OR TI=(scor*) OR TI=(instrument*) OR TI=(tool*) OR TI=(model) OR AB=(approach) OR AB=(assessment) OR AB=(scor*) OR AB=(instrument*) OR AB=(tool*) OR AB=(model)) AND (TI=(chronic kidney failure) OR TI=(chronic renal disease) OR TI=(CKD) OR TI=(chronic kidney disease) OR TI=(diabetic kidney disease) OR TI=(DKD) OR AB=(chronic kidney failure) OR AB=(chronic renal disease) OR AB=(CKD) OR AB=(chronic kidney disease) OR AB=(diabetic kidney disease) OR AB=(DKD)) AND (TI=(Klotho) OR AB=(Klotho) OR TI=(optical coherence tomography) OR AB=(optical coherence tomography) OR TI=(eye) OR AB=(eye) OR TI=(retina*) OR AB=(retina*) OR TI=(ocular) OR AB=(ocular) OR TI=(HbA1c) OR AB=(HbA1c) OR TI=(glycated hemoglobin) OR AB=(glycated hemoglobin) OR TI=(glycosylated hemoglobin) OR AB=(glycosylated hemoglobin))

| Platform | Web of Science | |
| --- | --- | --- |
|  |  |  |
| 1a | TI=(indic*) OR AB=(indic*) | 5,867,426 |
| 1b | TI=(predict*) OR AB=(predict*) | 3,557,073 |
| 1c | TI=(risk*) OR AB=(risk*) | 3,165,471 |
| 1d | TI=(prognos*) OR AB=(prognos*) | 734,335 |
| 1e | 1a OR 1b OR 1c OR 1d | 11,509,358 |
|  |  |  |
| 2a | TI=(model) OR AB=(model) | 8,462,025 |
| 2b | TI=(approach) OR AB=(approach) | 4,640,698 |
| 2c | TI=(assessment) OR AB=(assessment) | 1,819,842 |
| 2d | TI=(tool*) OR AB=(tool*) | 1,625,260 |
| 2e | TI=(scor*) OR AB=(scor*) | 1,384,770 |
| 2f | TI=(instrument*) OR AB=(instrument*) | 612,652 |
| 2g | 2a OR 2b OR 2c OR 2d OR 2e OR 2f | 14,947,590 |
|  |  |  |
| 3a | TI=(chronic kidney disease) OR AB=(chronic kidney disease) | 82,810 |
| 3b | TI=(chronic renal disease) OR AB=(chronic renal disease) | 52,268 |
| 3c | TI=(CKD) OR AB=(CKD) | 41,679 |
| 3d | TI=(chronic kidney failure) OR AB=(chronic kidney failure) | 16,542 |
| 3e | TI=(diabetic kidney disease) OR AB=(diabetic kidney disease) | 13,378 |
| 3f | TI=(DKD) OR AB=(DKD) | 2,317 |
| 3g | 3a OR 3b OR 3c OR 3d OR 3e OR 3f | 119,674 |
|  |  |  |
| 4a | TI=(eye) OR AB=(eye) | 421,157 |
| 4b | TI=(retina*) OR AB=(retina*) | 221,498 |
| 4c | TI=(ocular) OR AB=(ocular) | 130,21 |
| 4d | TI=(optical coherence tomography) OR AB=(optical coherence tomography) | 51,196 |
| 4e | TI=(HbA1c) OR AB=(HbA1c) | 33,759 |
| 4f | TI=(glycated hemoglobin) OR AB=(glycated hemoglobin) | 15,437 |
| 4g | TI=(glycosylated hemoglobin) OR AB=(glycosylated hemoglobin) | 9,954 |
| 4h | TI=(Klotho) OR AB=(Klotho) | 3,766 |
| 4i | 4a OR 4b OR 4c OR 4d OR 4e OR 4f OR 4g… OR 4h | 719,247 |
|  |  |  |
| 5a | 1e OR 2g OR 3g OR 4i | 700 |
|  |  |  |
| 6a | filter from 15.01.2019 | 526 |
