# Supplementary material for: Approaches to predict future type 2 diabetes mellitus and chronic kidney disease: A scoping review
Source: PLoS One. 2025 Jun 11;20(6):e0325182. doi: 10.1371/journal.pone.0325182 (PMC12157063; doi:10.1371/journal.pone.0325182)
Supplement: S3 Appendix — (DOCX) [file pone.0325182.s003.docx]

**S3 Appendix. Systematic reviews for T2DM that have been identified in step one and did not meet the inclusion criteria but indicate further approaches that have not been covered in step one.**

| **T2DM** |  |  |  |
| --- | --- | --- | --- |
| **Review author (year)** | **Prediction approach** | **Indication of future development and/or use** | **Related key words** |
| Que (2021) [1] | Gut microbiota | “By selecting bacterial features and building an RF model, we raise the possibility of a fecal bacterial mode of monitoring gut health and a complementary approach for risk assessment of T2DM.” | Microbiota, microbiome, microflora |
| Ramzan (2022) [2] | BCAA | “We suggest the potential utility of BCAAs as an early biomarker for T2DM irrespective of follow-up time. ” | Branched chain amino acids, BCAA |
| Roshanzamir (2018) [3] | Fetuin-A | “Fetuin-A may be a potential screening and prediction biomarker in T2D patients.” | Fetuin-A |
| Witt (2019) [4] | Continuous Glucose Monitoring | “CGMs also enable discovery of individual-specific dynamic glucose patterns and may also be informative for pre-diabetics or non-diabetics for identifying risk factors and trajectories of developing diabetes.” | Dexcom, Abbott, Medtronic, Senseonics, continuous glucose monitoring |

BCAA: branched chained amino-acids; CGM: continuous glucose monitoring; RF: random forest; T2D/T2DM: diabetes mellitus type II.

**References**

1. Que Y, Cao M, He J, Zhang Q, Chen Q, Yan C, et al. Gut Bacterial Characteristics of Patients With Type 2 Diabetes Mellitus and the Application Potential. Frontiers in immunology. 2021;12:722206. Epub 2021/09/07. doi: 10.3389/fimmu.2021.722206. PubMed PMID: 34484230; PubMed Central PMCID: PMCPMC8415158.

2. Ramzan I, Ardavani A, Vanweert F, Mellett A, Atherton PJ, Idris I. The Association between Circulating Branched Chain Amino Acids and the Temporal Risk of Developing Type 2 Diabetes Mellitus: A Systematic Review & Meta-Analysis. Nutrients. 2022;14(20). Epub 2022/10/28. doi: 10.3390/nu14204411. PubMed PMID: 36297095; PubMed Central PMCID: PMCPMC9610746.

3. Roshanzamir F, Miraghajani M, Rouhani MH, Mansourian M, Ghiasvand R, Safavi SM. The association between circulating fetuin-A levels and type 2 diabetes mellitus risk: systematic review and meta-analysis of observational studies. Journal of endocrinological investigation. 2018;41(1):33-47. Epub 2017/06/24. doi: 10.1007/s40618-017-0697-8. PubMed PMID: 28643299.

4. Witt D, Kellogg R, Snyder M, Dunn J. Windows Into Human Health Through Wearables Data Analytics. Current opinion in biomedical engineering. 2019;9:28-46. Epub 2019/12/14. doi: 10.1016/j.cobme.2019.01.001. PubMed PMID: 31832566; PubMed Central PMCID: PMCPMC6907085.
