# Supplementary material for: Approaches to predict future type 2 diabetes mellitus and chronic kidney disease: A scoping review
Source: PLoS One. 2025 Jun 11;20(6):e0325182. doi: 10.1371/journal.pone.0325182 (PMC12157063; doi:10.1371/journal.pone.0325182)
Supplement: S4 Appendix — (DOCX) [file pone.0325182.s004.docx]

**S4 Appendix. Study characteristics of identified literature about prediction approaches for T2DM**

**T2DM – Systematic Reviews**

| **Author(s)** | **Year of publication** | **Country of origin** | **Aims/Purpose** |
| --- | --- | --- | --- |
| Abbasi [1] | 2012 | Netherlands | To identify and validate prediction models for the risk of developing T2DM |
| Asgari [2] | 2021 | Iran | “To provide an overview of prediction models of” U-T2DM or I-T2DM” |
| Bao [3] | 2013 | United States | “To evaluate the predictive performance of genetic risk models based on risk loci identified and/or confirmed in genome-wide association studies for” T2DM |
| Buijsse [4] | 2011 | Germany | To identify T2DM risk scores for general populations and to assess transferability to other cohorts |
| Collins [5] | 2011 | United Kingdom | To review and assess the methodological conduct of risk prediction models for undiagnosed or future T2DM |
| Ekure [6] | 2022 | Sub-Saharan Africa | To review “studies on diabetes risk assessment tools used in sub-Saharan Africa (SSA) to diagnose diabetes in symptomatic and asymptomatic patients” |
| Fregoso‑Aparici [7] | 2021 | Mexico | To identify techniques and features of machine learning models for the prediction of T2DM |
| Hu [8] | 2016 | Singapore | To identify common risk factors in T2DM risk scores with high discrimination, as well as factors with high influence on risk scores in Asian populations and to propose a set of factors applicable to Singapore's multi-ethnic population |
| Nawi [9] | 2022 | Malaysia | “To outline the relative performance accuracies in predicting prediabetes conditions in different machine learning algorithms” |
| Nguyen [10] | 2012 | Australia | “To evaluate the components and validity of risk scores for the screening and early detection of” T2DM |
| Noble [11] | 2011 | United Kingdom | “To evaluate current risk models and scores for” T2DM “and inform selection and implementation of these in practice” |
| Nusrianto [12] | 2019 | Indonesia | To assess “whether VAI can be used as a predictor of T2DM in Asian population with different body composition compared to the Caucasian population” |
| Padilla-Martinez [13] | 2020 | Poland | To review studies comparing the accuracy of polygenic risk scores developed during the last two decades |
| Silva [14] | 2020 | Australia | “To assess the use and predictive performance of ML models for T2DM prediction in community settings” |
| Waugh [15] | 2007 | United Kingdom | To assess aims, indications and clinical and economic effectiveness of screening for T2DM to inform policy measures. |
| Willis [16] | 2014 | United Kingdom | To review the existing literature assessing community pharmacy-based screening for risk factors for diabetes and individuals with a high cardiovascular disease risk |
| Xu [17] | 2022 | China | “To evaluate the performance of published T2DM risk prediction models in Chinese people with IH to inform them about the choice of primary diabetes prevention measures” |
| Yonel [18] | 2020 | United Kingdom | “To review evidence associated with the use of primary care dental services for the identification of undiagnosed hyperglycemia or” T2DM in adults |
| Yoshizawa [19] | 2016 | Japan | “To assess the predictive ability of non-blood-based risk assessment for future incident T2DM” |

IH: intermediate hyperglycemia; SSA: sub-Saharan Africa; T2DM: diabetes mellitus type II; I-T2DM: incident diabetes mellitus type II; U-T2DM: undiagnosed diabetes mellitus type II; VAI: Visceral Adiposity Index.

**T2DM – Primary literature**

| **Author(s)** | **Year of publication** | **Country of origin** | **Aims/Purpose** | **Population and sample size within the source of evidence (if applicable)** | **Methods** | **Outcomes and details (e.g., how measured) (if applicable)** |
| --- | --- | --- | --- | --- | --- | --- |
| Aasmets [20] | 2021 | Estonia | To test the predictive potential of the gut microbiome for T2DM development during the healthy and prediabetic disease stages | 608 randomly selected men from eastern Finland (45-73 years; including different metabolic traits such as T2D, hypertension, and obesity; subset from the METSIM follow-up study)  Investigation of stool samples taken at three time points (baseline, 18-month follow-up, and 48-month follow-up) | *Longitudinal study*  “A comprehensive machine learning strategy was implemented to identify microbial biomarkers and their effect on numerous metabolic traits”  “independent prospective models were trained for the 18-month and 48-month follow-up period” and “models including microbial predictors were compared to models excluding microbial predictors”  “accumulated local effects methodology was used to plot the effect of the microbial biomarkers for predicting the corresponding metabolic trait” | “The identified microbiome biomarkers provide a predictive measure for various metabolic  traits related to” T2DM, “thus providing an additional parameter for personal risk assessment”  The “work also highlights the need for robust modeling strategies and the  value of interpretable machine learning” |
| Chen [21] | 2016a | China | “To evaluate the potential role of BCAAs and AAAs in predicting the diabetes development in Chinese populations” | *Longitudinal study*  213 healthy individuals (20–75 years)  *Cross-sectional study*  216 Chinese individuals (20–65 years) including healthy lean, healthy overweight or obese, and overweight or obese with T2DM from the SHOS,  72 participants (36 males, 36 females) in each of the three groups | *Longitudinal study*  Assessment of baseline levels of five AAs in patients with the risk of developing diabetes in an average of 10 years  *Cross-sectional study*  cross-sectional comparison of serum levels of five AAs in metabolically healthy or unhealthy patients | The “findings verified the close correlation of BCAAs and AAAs with insulin resistance and future development of diabetes in Chinese populations and highlighted the predictive value of these markers for future development of” DM |
| Chen [22] | 2016b | China | To assess the association of the AA tryptophan and T2DM development and to evaluate its performance with already existing AA predictors for incident T2DM | 213 healthy individuals (20–75 years) with normal glucose tolerance selected from the SHDS | *Prospective cohort study*  “The 5AAs- and 6AAs- combined scores were generated by linear regression based on the abundance  of 5 or 6 amino acids of interest”  “logistic regression models were performed”  “The predictive performance of the combined score improved after taking tryptophan  into account“ | The “findings unveiled the potential of tryptophan as a new marker associated with diabetes risk in Chinese populations. The addition of tryptophan provided complementary  value to the existing” AA predictors |
| Flores-Guerrero [23] | 2020 | Netherlands | To evaluate the ability of the DRI to predict incident T2DM in a large adult population | 6,134 nondiabetic men and women from the PREVEND study cohort (2001-2003) | *Prospective cohort study*  DRI scores developed by combining the LP-IR (including 6 lipoprotein subspecies and size parameters) and BCAAs | Association of Higher DRI scores and an increased risk of T2DM, independent of clinical risk factors for T2DM |
| Lu [24] | 2016 | Singapore | “To identify metabolic signatures associated with an increased risk of” T2DM by characterizing human serum metabolic profiles | 197 randomly selected individuals with newly diagnosed DM, cardiovascular disease or cancer at blood collection from the SCHS (63,257 Chinese men and women (45–74 years) in Singapore) | *Nested case–control study*  “Baseline serum metabolite profiles were measured using LC-MS and GC-MS during a 6-year follow-up” | The “findings show that BCAA and NEFA are potent predictors of diabetes development in Chinese adults”  The “results also indicate the potential of lysophospholipids for predicting diabetes” |
| Sawicki [25] | 2023 | United States | To analyse longitudinal patterns of change in BCAAs to enhance the understanding of the pathophysiology of insulin resistance, to guide preventive interventions in young individuals at high risk for developing DM | 3,081 participants from the CARDIA study cohort (black and white men and women (18–30 years), recruited from 1985–1986 across 4 urban US sites) | *Longitudinal study*  Measurement of BCAAs over 28 years, Trajectories of circulating BCAA concentrations from years 2–30 (for prevalent DM) or years 2–20 (for incident DM) were determined by latent class modeling | “BCAA levels track over a 28-year span in most young adults, but serial clinical metabolomic measurements identify subpopulations with rising levels associated with high risk of DM in later life” |

AA: amino acid,; AAA: aromatic amino acids; BCAA: branched-chain amino acid; CARDIA: Coronary Artery Risk Development in Young Adults; DM: diabetes mellitus; DRI: Diabetes Risk Index; GC-MS: Gas Chromatography Mass Spectrometry; LC-MS: Liquid Chromatography Mass Spectrometry; LP-IR: Lipoprotein Insulin Resistance Index; METSIM: Metabolic Syndrome in Men study; NEFA: non esterified fatty acids; PREVEND: Prevention of Renal and Vascular End-Stage Disease; T2DM: diabetes mellitus type II; SCHS: Singapore Chinese Health Study; SHDS: Shanghai Diabetes Study; SHOS: Shanghai Obesity Study.

**T2DM – Structured search**

| **Source** | **Year of publication** | **Name of the approach** | **Description** |
| --- | --- | --- | --- |
| Medtech Insight, (NuraLogix Corp., Toronto [26] | 2022 | - | Smartphone app which predicts the risk for pre-diabetes with a 30-second video selfie. App uses a novel imaging method called transdermal optical imaging. |
| IBS – Intelligent Bio Solutions [27] | 2022 | - | Sensor that measures glucose in saliva. Sensor is developed as a point of care self-test for persons with diabetes and currently not suitable as an addition to blood glucose monitoring for deciding on diabetes treatment. |
| University of Edinburgh,  [28] | 2023 | - | Predict the ten-year risk of developing T2DM by inclusion of DNA methylation data alongside classical risk factors, such as sex, age, and BMI, |
| School of Public Health at the University of Minnesota  [29] | 2019 | - | Subgingival bacteria were linked to changes in future glucose levels. The resulting microbial ‘dysbiosis score’ was found to be a stronger predictor of rising glucose levels than age or obesity. |

BMI: body mass index; DKD: diabetic kidney disease; DNA: deoxyribonucleic acid; T2DM: diabetes mellitus type II.

**References**

1. Abbasi A, Peelen LM, Corpeleijn E, van der Schouw YT, Stolk RP, Spijkerman AM, et al. Prediction models for risk of developing type 2 diabetes: systematic literature search and independent external validation study. BMJ. 2012;345:e5900. Epub 2012/09/20. doi: 10.1136/bmj.e5900. PubMed PMID: 22990994; PubMed Central PMCID: PMCPMC3445426.

2. Asgari S, Khalili D, Hosseinpanah F, Hadaegh F. Prediction Models for Type 2 Diabetes Risk in the General Population: A Systematic Review of Observational Studies. International journal of endocrinology and metabolism. 2021;19(3):e109206. Epub 2021/09/28. doi: 10.5812/ijem.109206. PubMed PMID: 34567135; PubMed Central PMCID: PMCPMC8453657.

3. Bao W, Hu FB, Rong S, Rong Y, Bowers K, Schisterman EF, et al. Predicting risk of type 2 diabetes mellitus with genetic risk models on the basis of established genome-wide association markers: a systematic review. American journal of epidemiology. 2013;178(8):1197-207. Epub 2013/09/07. doi: 10.1093/aje/kwt123. PubMed PMID: 24008910; PubMed Central PMCID: PMCPMC3792732.

4. Buijsse B, Simmons RK, Griffin SJ, Schulze MB. Risk assessment tools for identifying individuals at risk of developing type 2 diabetes. Epidemiologic reviews. 2011;33(1):46-62. Epub 2011/05/31. doi: 10.1093/epirev/mxq019. PubMed PMID: 21622851; PubMed Central PMCID: PMCPMC3132807.

5. Collins GS, Mallett S, Omar O, Yu LM. Developing risk prediction models for type 2 diabetes: a systematic review of methodology and reporting. BMC medicine. 2011;9:103. Epub 2011/09/10. doi: 10.1186/1741-7015-9-103. PubMed PMID: 21902820; PubMed Central PMCID: PMCPMC3180398.

6. Ekure E, Ovenseri-Ogbomo G, Osuagwu UL, Agho KE, Ekpenyong BN, Ogbuehi KC, et al. A systematic review of diabetes risk assessment tools in sub-Saharan Africa. Int J Diabetes Dev Ctries. 2022;42(3):380-93. doi: 10.1007/s13410-022-01045-8.

7. Fregoso-Aparicio L, Noguez J, Montesinos L, García-García JA. Machine learning and deep learning predictive models for type 2 diabetes: a systematic review. Diabetology & metabolic syndrome. 2021;13(1):148. Epub 2021/12/22. doi: 10.1186/s13098-021-00767-9. PubMed PMID: 34930452; PubMed Central PMCID: PMCPMC8686642.

8. Hu PL, Koh YLE, Tan NC. The utility of diabetes risk score items as predictors of incident type 2 diabetes in Asian populations: An evidence-based review. Diabetes research and clinical practice. 2016;122:179-89. doi: 10.1016/j.diabres.2016.10.019.

9. Nawi AM, Kamaruddin P, Nordin NRM, Soffian SSS, Baharom M. Machine Learning Models in Prediabetes Screening: A Systematic Review. J Clin Diagn Res. 2022;16(5). doi: 10.7860/JCDR/2022/53411.16385. PubMed PMID: WOS:000803032000001.

10. Nguyen CT, Lee AH, Binns CW. Components and validity of risk scores for screening for Type 2 diabetes mellitus. Diabetes Manage. 2012;2(5):419-25. doi: 10.2217/dmt.12.43.

11. Noble D, Mathur R, Dent T, Meads C, Greenhalgh T. Risk models and scores for type 2 diabetes: systematic review. BMJ. 2011;343:d7163. Epub 2011/11/30. doi: 10.1136/bmj.d7163. PubMed PMID: 22123912; PubMed Central PMCID: PMCPMC3225074.

12. Nusrianto R, Tahapary DL, Soewondo P. Visceral adiposity index as a predictor for type 2 diabetes mellitus in Asian population: A systematic review. Diabetes & metabolic syndrome. 2019;13(2):1231-5. Epub 2019/07/25. doi: 10.1016/j.dsx.2019.01.056. PubMed PMID: 31336469.

13. Padilla-Martínez F, Collin F, Kwasniewski M, Kretowski A. Systematic Review of Polygenic Risk Scores for Type 1 and Type 2 Diabetes. International journal of molecular sciences. 2020;21(5). Epub 2020/03/07. doi: 10.3390/ijms21051703. PubMed PMID: 32131491; PubMed Central PMCID: PMCPMC7084489.

14. Silva K, Lee WK, Forbes A, Demmer RT, Barton C, Enticott J. Use and performance of machine learning models for type 2 diabetes prediction in community settings: A systematic review and meta-analysis. International journal of medical informatics. 2020;143:104268. Epub 2020/09/21. doi: 10.1016/j.ijmedinf.2020.104268. PubMed PMID: 32950874.

15. Waugh N, Scotland G, McNamee P, Gillett M, Brennan A, Goyder E, et al. Screening for type 2 diabetes: Literature review and economic modelling. Health Technol Assess. 2007;11(17):iii-106. doi: 10.3310/hta11170.

16. Willis A, Rivers P, Gray LJ, Davies M, Khunti K. The effectiveness of screening for diabetes and cardiovascular disease risk factors in a community pharmacy setting. PloS one. 2014;9(4). doi: 10.1371/journal.pone.0091157.

17. Xu SS, Coleman RL, Wan Q, Gu YQ, Meng G, Song K, et al. Risk prediction models for incident type 2 diabetes in Chinese people with intermediate hyperglycemia: a systematic literature review and external validation study. Cardiovascular diabetology. 2022;21(1). doi: 10.1186/s12933-022-01622-5. PubMed PMID: WOS:000853436400001.

18. Yonel Z, Cerullo E, Kröger AT, Gray LJ. Use of dental practices for the identification of adults with undiagnosed type 2 diabetes mellitus or non-diabetic hyperglycaemia: a systematic review. Diabetic medicine : a journal of the British Diabetic Association. 2020;37(9):1443-53. Epub 2020/05/20. doi: 10.1111/dme.14324. PubMed PMID: 32426909.

19. Yoshizawa S, Kodama S, Fujihara K, Ishiguro H, Ishizawa M, Matsubayashi Y, et al. Utility of nonblood-based risk assessment for predicting type 2 diabetes mellitus: A meta-analysis. Prev Med. 2016;91:180-7. doi: <https://doi.org/10.1016/j.ypmed.2016.07.026>.

20. Aasmets O, Lüll K, Lang JM, Pan C, Kuusisto J, Fischer K, et al. Machine Learning Reveals Time-Varying Microbial Predictors with Complex Effects on Glucose Regulation. mSystems. 2021;6(1). Epub 2021/02/18. doi: 10.1128/mSystems.01191-20. PubMed PMID: 33594006; PubMed Central PMCID: PMCPMC8573957.

21. Chen T, Ni Y, Ma X, Bao Y, Liu J, Huang F, et al. Branched-chain and aromatic amino acid profiles and diabetes risk in Chinese populations. Sci Rep. 2016a;6:20594. Epub 2016/02/06. doi: 10.1038/srep20594. PubMed PMID: 26846565; PubMed Central PMCID: PMCPMC4742847.

22. Chen T, Zheng X, Ma X, Bao Y, Ni Y, Hu C, et al. Tryptophan Predicts the Risk for Future Type 2 Diabetes. PloS one. 2016b;11(9):e0162192. Epub 2016/09/07. doi: 10.1371/journal.pone.0162192. PubMed PMID: 27598004; PubMed Central PMCID: PMCPMC5012675.

23. Flores-Guerrero JL, Gruppen EG, Connelly MA, Shalaurova I, Otvos JD, Garcia E, et al. A Newly Developed Diabetes Risk Index, Based on Lipoprotein Subfractions and Branched Chain Amino Acids, is Associated with Incident Type 2 Diabetes Mellitus in the PREVEND Cohort. Journal of clinical medicine. 2020;9(9). Epub 2020/09/02. doi: 10.3390/jcm9092781. PubMed PMID: 32867285; PubMed Central PMCID: PMCPMC7563197.

24. Lu Y, Wang Y, Ong CN, Subramaniam T, Choi HW, Yuan JM, et al. Metabolic signatures and risk of type 2 diabetes in a Chinese population: an untargeted metabolomics study using both LC-MS and GC-MS. Diabetologia. 2016;59(11):2349-59. Epub 2016/08/16. doi: 10.1007/s00125-016-4069-2. PubMed PMID: 27514531.

25. Sawicki KT, Ning H, Allen NB, Carnethon MR, Wallia A, Otvos JD, et al. Longitudinal trajectories of branched chain amino acids through young adulthood and diabetes in later life. JCI insight. 2023;8(8). Epub 2023/04/24. doi: 10.1172/jci.insight.166956. PubMed PMID: 37092552; PubMed Central PMCID: PMCPMC10243737.

26. Webb M. HLTH 2022: NuraLogix Demo AI Models That Can Predict Risk For Pre-Diabetes: Medtech Insight; 2022 [21.06.2023]. Available from: <https://medtech.pharmaintelligence.informa.com/MT146039/HLTH-2022-NuraLogix-Demo-AI-Models-That-Can-Predict-Risk-For-Pre-Diabetes>.

27. Intelligent Bio Solutions. The Saliva Glucose Biosensor 2022 [14.06.2022]. Available from: <https://ibs.inc/the-saliva-glucose-biosensor/#:~:text=The%20Saliva%20Glucose%20Biosensor%20is,glucose%20in%20saliva%2C%20not%20blood>.

28. Cheng Y, Gadd DA, Gieger C, Monterrubio-Gómez K, Zhang Y, Berta I, et al. Development and validation of DNA methylation scores in two European cohorts augment 10-year risk prediction of type 2 diabetes. Nat Aging. 2023;3(4):450-8. doi: 10.1038/s43587-023-00391-4.

29. Plain C. Oral bacteria may reveal risk for diabetes and cardiovascular disease Minneapolis: School of Public Health. University of Minnesota; 2019 [15.06.2023]. Available from: <https://www.sph.umn.edu/news/oral-bacteria-may-reveal-risk-for-diabetes-and-cardiovascular-disease/>.
