# Supplementary material for: Approaches to predict future type 2 diabetes mellitus and chronic kidney disease: A scoping review
Source: PLoS One. 2025 Jun 11;20(6):e0325182. doi: 10.1371/journal.pone.0325182 (PMC12157063; doi:10.1371/journal.pone.0325182)
Supplement: S5 Appendix — (DOCX) [file pone.0325182.s005.docx]

**S5 Appendix. Systematic reviews for CKD that have been identified in step one and did not meet the inclusion criteria but indicate further approaches that have not been covered in step one.**

| **CKD** |  |  |  |
| --- | --- | --- | --- |
| **Review author (year)** | **Prediction approach** | **Indication of future development and/or use** | **Related key words** |
| Liu (2019) [1] | Klotho | “Levels of sKlotho are positively correlated with the eGFR, and low sKlotho levels are significantly associated with an increased risk of poor kidney outcomes. Therefore, sKlotho could be used as a novel biomarker for early diagnosis and prognostic assessment for patients with chronic kidney disease.” | Klotho, alpha-Klotho, α-Klotho |
| Peng (2022) [2] | Artificial Intelligence - Ocular Images | “In the study predicting both CKD and T2DM, a lower survival probability was found between the high-risk and other groups stratified from the DL predictor features. […] Altogether, the scores generated from extracted features of the ocular image, similar to the imaging marker seen in cancer studies, can provide early insight into systemic diseases prognosis.” | Optical coherence tomography, external eye photo, dynamic retina vessel analysis, ocular images |
| Qu (2022) [3] | HbA1c variability | “People with the highest quantile of HbA1c-CV were associated with increased risks of kidney failure, progression to CKD, […] A careful review of historical HbA1c measures may facilitate clinicians to identify patients at high risks. However, also it remains necessary to explore potential clinical implementation strategies for these HbA1c variability parameters in people with type 2 diabetes.” | HbA1c, glycated hemoglobin, glycosylated haemoglobin, |

CKD: chronic kidney disease; DL: deep learning; HbA1c: glycated hemoglobin; HbA1c-CV: glycated hemoglobin coefficient of variation; sKlotho: soluble or secreted Klotho; T2DM: diabetes mellitus type II.

**References**

1. Liu QF, Yu LX, Feng JH, Sun Q, Li SS, Ye JM. The Prognostic Role of Klotho in Patients with Chronic Kidney Disease: A Systematic Review and Meta-analysis. Disease Markers. 2019;6468729. doi: 10.1155/2019/6468729.

2. Peng Q, Tseng R, Tham YC, Cheng CY, Rim TH. Detection of Systemic Diseases From Ocular Images Using Artificial Intelligence: A Systematic Review. Asia-Pacific journal of ophthalmology (Philadelphia, Pa). 2022;11(2):126-39. Epub 2022/05/10. doi: 10.1097/apo.0000000000000515. PubMed PMID: 35533332.

3. Qu F, Shi Q, Wang Y, Shen Y, Zhou K, Pearson ER, et al. Visit-to-visit glycated hemoglobin A1c variability in adults with type 2 diabetes: a systematic review and meta-analysis. Chinese medical journal. 2022;135(19):2294-300. Epub 2022/08/12. doi: 10.1097/cm9.0000000000002073. PubMed PMID: 35952315; PubMed Central PMCID: PMCPMC9771337.
