# Supplementary material for: Approaches to predict future type 2 diabetes mellitus and chronic kidney disease: A scoping review
Source: PLoS One. 2025 Jun 11;20(6):e0325182. doi: 10.1371/journal.pone.0325182 (PMC12157063; doi:10.1371/journal.pone.0325182)
Supplement: S6 Appendix — (DOCX) [file pone.0325182.s006.docx]

**S6 Appendix. Study characteristics of identified literature about prediction approaches for CKD**

**CKD – Systematic Reviews**

| **Author(s)** | **Year of publication** | **Country of origin** | **Aims/Purpose** |
| --- | --- | --- | --- |
| Aparcana-Granda [1] | 2022 | Peru | “To summarise available CKD diagnostic and prognostic models in LIMCs” |
| Collins [2] | 2013 | United Kingdom | To assess “studies developing risk prediction models for CKD or progression to end-stage kidney failure” |
| Echouffo-Tcheugui [3] | 2012 | United States | To assess risk models for the prediction of CKD and its progression and to evaluate their suitability for clinical use |
| Fraccaro [4] | 2016 | United Kingdom | To validate and compare previously published models for predicting 5-year CKD risk by using routine healthcare records from a UK population |
| Saputro [5] | 2021 | Indonesia | To summarise prognostic models for diabetic microvascular complications and to assess their performance in prediction of complications |
| Slieker [6] | 2021 | Netherlands | To identify and assess the quality, accuracy, and external validity of prognostic models for nephropathy in people with T2DM |

CKD: chronic kidney disease; T2DM: diabetes mellitus type II; UK: United Kingdom; LIMCs: low-income and middle-income countries.

**CKD – Primary literature**

| **Author(s)** | **Year of publication** | **Country of origin** | **Aims/Purpose** | **Population and sample size within the source of evidence (if applicable)** | **Methods** | **Outcomes and details (e.g., how measured) (if applicable)** |
| --- | --- | --- | --- | --- | --- | --- |
| Xin [7] | 2023 | China | To investigate the association between DKD and HGI in Chinese T2DM patients,  To construct a risk score to predict a person’s risk of DKD based on HGI | 1,622 patients with T2DM (1,016 males and 606 females, average age: 55.8, average duration of T2DM: 9.31 years) divided into 1,232 cases of non-DKD and 390 cases of DKD | *Cross-sectional study*  Retrospective analysis of T2DM patients (low HGI, medium HGI, high HGI) to analyse occurrence of DKD, Multivariate logistics regression analysis to analyse the risk factors of DKD in patients with T2DM | High HGI is found to be associated with an increased risk of developing DKD. The DKD risk score may be used as one of the risk predictors of DKD in T2DM populations |
| Yan [8] | 2022 | Japan | To clarify the relationship between risk of DKD and visit-to-visit variability of HbA1c,  to identify the most useful index of visit-to-visit variability of HbA1c | 728 records of consecutive T2DM patients (1999-2019), inclusion of 699 subjects (477 males, 222 females) | *Retrospective longitudinal study* Diagnostic criteria based on HbA1c, typical symptoms of chronic hyperglycaemia, values of plasma glucose, and/or a prior DM diagnosis,  associations between indices and the development/progression of DKD were examined | “Visit-to-visit variability of HbA1c was an independent risk factor of microalbuminuria in association with oxidative stress among T2DM patients. HbA1c-AUC, may be a potent prognostic indicator in predicting the risk of microalbuminuria” |

DKD: diabetic kidney disease; HbA1c: glycated haemoglobin; HbA1c-AUC: HbA1c-area under the curve; HGI: haemoglobin glycation index; T2DM: diabetes mellitus type II.

**CKD – Structured search**

| **Source** | **Year of publication** | **Name of the approach** | **Description** |
| --- | --- | --- | --- |
| Chuang [9] | 2020 | SUN-156 DNlite-IVD103 | Prospective cohort study demonstrating that the DNlite-IVD103 test (a novel urinary test, detecting a DKD biomarker) for identifying progressive decliners of renal function in T2DM with microalbuminuria can be used as risk assessment for predicting short term renal function change. |
|  |  |  |  |
| Hu [11] | 2020 | DN incidence risk nomogram | Study on developing a novel nomogram to predict DN incidence risk among T2DM patients using eight variables (such as demographics, lifestyle habits, physical examination results and biochemical test results). |
| Li [12] | 2018 | Quality-of-care scoring system | Retrospective cohort study on developing a long-term quality-of care score (from 0 to 8) for predicting the occurrence of CKD in patients with T2DM, using process indicators (such as frequencies of HbA1c, lipid profile testing, urine, foot, and retinal examinations), intermediate outcome indicators (low-density lipoprotein, blood pressure, and HbA1c) and comorbidity of hypertension. |
| Ma [13] | 2017 | Genetic Risk Score | Creation of a GRS using 53 SNPs associated with creatinine-based eGFR (16 known and 37 novel SNPs); investigation whether the updated GRS can predict an increased risk of stage 3 CKD events, independent of general clinical risk factors. |
| Peters [14] | 2017 | PromarkerD | Discovery of plasma markers building the PromarkerD, a novel diagnostic test combining a panel of plasma biomarkers (apoA4, CD5L and IGFBP3) and clinical variables (age, HDL-cholesterol and eGFR) to accurately predict future renal decline in people with T2DM. |

apoA4: apolipoprotein A-IV; CD5L: CD5 antigen-like; DKD: diabetic kidney disease; DN: diabetic nephropathy; eGFR: estimated glomerular filtration rate; GRS: genetic risk score; HbA1c: glycated haemoglobin; HDL: high-density lipoprotein; IGFBP3: insulin-like growth factor-binding protein 3; SNPs: single nucleotide polymorphisms; T2DM: diabetes mellitus type II.

**References**

1. Aparcana-Granda DJ, Ascencio EJ, Carrillo Larco RM. Systematic review of diagnostic and prognostic models of chronic kidney disease in low-income and middle-income countries. BMJ open. 2022;12(3):e058921. Epub 2022/03/17. doi: 10.1136/bmjopen-2021-058921. PubMed PMID: 35292503; PubMed Central PMCID: PMCPMC8928240.

2. Collins GS, Omar O, Shanyinde M, Yu LM. A systematic review finds prediction models for chronic kidney disease were poorly reported and often developed using inappropriate methods. Journal of clinical epidemiology. 2013;66(3):268-77. Epub 2012/11/03. doi: 10.1016/j.jclinepi.2012.06.020. PubMed PMID: 23116690.

3. Echouffo-Tcheugui JB, Kengne AP. Risk models to predict chronic kidney disease and its progression: a systematic review. PLoS medicine. 2012;9(11):e1001344. Epub 2012/11/28. doi: 10.1371/journal.pmed.1001344. PubMed PMID: 23185136; PubMed Central PMCID: PMCPMC3502517.

4. Fraccaro P, van der Veer S, Brown B, Prosperi M, O'Donoghue D, Collins GS, et al. An external validation of models to predict the onset of chronic kidney disease using population-based electronic health records from Salford, UK. BMC medicine. 2016;14:104. Epub 2016/07/13. doi: 10.1186/s12916-016-0650-2. PubMed PMID: 27401013; PubMed Central PMCID: PMCPMC4940699.

5. Saputro SA, Pattanaprateep O, Pattanateepapon A, Karmacharya S, Thakkinstian A. Prognostic models of diabetic microvascular complications: a systematic review and meta-analysis. Syst Rev. 2021;10(1):288. doi: 10.1186/s13643-021-01841-z.

6. Slieker RC, van der Heijden AAWA, Siddiqui MK, Langendoen-Gort M, Nijpels G, Herings R, et al. Performance of prediction models for nephropathy in people with type 2 diabetes: systematic review and external validation study. BMJ. 2021;374:n2134. doi: 10.1136/bmj.n2134.

7. Xin S, Zhao X, Ding J, Zhang X. Association between hemoglobin glycation index and diabetic kidney disease in type 2 diabetes mellitus in China: A cross- sectional inpatient study. Frontiers in endocrinology. 2023;14:1108061. Epub 2023/03/28. doi: 10.3389/fendo.2023.1108061. PubMed PMID: 36967789; PubMed Central PMCID: PMCPMC10031087.

8. Yan Y, Kondo N, Oniki K, Watanabe H, Imafuku T, Sakamoto Y, et al. Predictive Ability of Visit-to-Visit Variability of HbA1c Measurements for the Development of Diabetic Kidney Disease: A Retrospective Longitudinal Observational Study. Journal of diabetes research. 2022;2022:6934188. Epub 2022/02/02. doi: 10.1155/2022/6934188. PubMed PMID: 35103243; PubMed Central PMCID: PMCPMC8800606.

9. Chuang LM, Lin WY, Huang CH, Lin CH, Tseng TL. SUN-156 DNlite-IVD103, a novel urinary test, predicts progressive eGFR decline in type 2 diabetes with microalbuminuria. Kidney Int Rep. 2020;5:S264. doi: 10.1016/j.ekir.2020.02.685.

11. Hu Y, Shi R, Mo R, Hu F. Nomogram for the prediction of diabetic nephropathy risk among patients with type 2 diabetes mellitus based on a questionnaire and biochemical indicators: a retrospective study. Aging. 2020;12(11):10317-36. Epub 2020/06/03. doi: 10.18632/aging.103259. PubMed PMID: 32484786; PubMed Central PMCID: PMCPMC7346021.

12. Li PI, Wang JN, Guo HR. Long-term quality-of-care score predicts incident chronic kidney disease in patients with type 2 diabetes. Nephrology, dialysis, transplantation : official publication of the European Dialysis and Transplant Association - European Renal Association. 2018;33(11):2012-9. Epub 2018/02/21. doi: 10.1093/ndt/gfx375. PubMed PMID: 29462347.

13. Ma J, Yang Q, Hwang SJ, Fox CS, Chu AY. Genetic risk score and risk of stage 3 chronic kidney disease. BMC nephrology. 2017;18(1):32. Epub 2017/01/21. doi: 10.1186/s12882-017-0439-3. PubMed PMID: 28103844; PubMed Central PMCID: PMCPMC5248454.

14. Peters KE, Davis WA, Ito J, Winfield K, Stoll T, Bringans SD, et al. Identification of Novel Circulating Biomarkers Predicting Rapid Decline in Renal Function in Type 2 Diabetes: The Fremantle Diabetes Study Phase II. Diabetes care. 2017;40(11):1548-55. doi: 10.2337/dc17-0911.
