# Supplementary material for: Approaches to predict future type 2 diabetes mellitus and chronic kidney disease: A scoping review
Source: PLoS One. 2025 Jun 11;20(6):e0325182. doi: 10.1371/journal.pone.0325182 (PMC12157063; doi:10.1371/journal.pone.0325182)
Supplement: S7 Appendix — (DOCX) [file pone.0325182.s007.docx]

**Appendix 7. Additional information on the identified approaches.**

| **T2DM** |  |  |  |  |  |  |
| --- | --- | --- | --- | --- | --- | --- |
| **Author (year)** | **Prediction approach** | **Sample size** | **Validation** | **Updates** | **Published after Tripod?** | **Applied in practice?** |
| [Conn (1940)](https://www.sciencedirect.com/science/article/abs/pii/S0025712516358357) [1] | Oral glucose tolerance test | N/A | Used for over 100 years and first described by Conn (1940) ([Bogdanet et al. 2020](https://www.ncbi.nlm.nih.gov/pmc/articles/PMC7693369/pdf/jcm-09-03451.pdf) [2])  Validity has recently been questioned ([De Sanctis et al. 2022](https://www.ncbi.nlm.nih.gov/pmc/articles/PMC9686143/) [3]) | N/A | No | Yes, used for prediction of T2DM ([Peddinti et al. 2019](https://www.ncbi.nlm.nih.gov/pmc/articles/PMC6382453/) [4]) |
| [Lindström (2003)](https://diabetesjournals.org/care/article/26/3/725/29197/The-Diabetes-Risk-ScoreA-practical-tool-to-predict) [5] | FINDRISC | N=4435 | Jølle et al. (2019) [6] | Not found | No | Recommended in guidelines [Jølle et al. (2019)](https://www.ncbi.nlm.nih.gov/pmc/articles/PMC6887494/#:~:text=In%20a%20Norwegian%20population%20followed,within%20the%20next%2010%20years.) [6] |
| [Kaczorowski (2009)](file://\\esfomedapp\Allgemein\Öffentlich\Projekte\BI%20-%20Scoping%20Review_T2DM%20+%20CKD\5.%20Endergebnisse\Überarbeitung%20Manuskript%20August%202024\Kaczorowski%20J,%20Robinson%20C,%20Nerenberg%20K.%20Development%20of%20the%20CANRISK%20questionnaire%20to%20screen%20for%20prediabetes%20and%20undiagnosed%20type%202%20diabetes.%20Can%20J%20Diabetes.%202009;33(4):381-5.%20doi:%2010.1016\S1499-2671(09)34008-3.) [7] | CANRISK |  | [Robinson (2011)](https://www.canada.ca/content/dam/phac-aspc/migration/phac-aspc/publicat/hpcdp-pspmc/32-1/assets/pdf/vol32n1-ar04-eng.pdf) [8] | Not found | No | Yes. Recommended by Canadian Task Force on Preventive Health care, together with FINDRISC ([canadiantaskforce](https://canadiantaskforce.ca/guidelines/published-guidelines/type-2-diabetes/) [9]) |
| [Chen (2010)](https://pubmed.ncbi.nlm.nih.gov/20170456/) [10] | AUSDRISK | N=6060 | [Chen (2010)](https://pubmed.ncbi.nlm.nih.gov/20170456/) [10] | Not found | No | Commonly used in clinical practice ([Buss 2021](https://www.sciencedirect.com/science/article/pii/S2211335521003387) [11]) |
| [Schulze (2007)](https://pubmed.ncbi.nlm.nih.gov/17327313/) [12] | German Diabetes Risk Score | N=25167 | [Mühlenbruch (2014)](https://www.sciencedirect.com/science/article/abs/pii/S0168822714001697) [13]  [Paprott](https://www.ncbi.nlm.nih.gov/pmc/articles/PMC5128853/pdf/bmjdrc-2016-000280.pdf) (2016) [14] | Yes, [Mühlenbruch (2014)](https://www.sciencedirect.com/science/article/abs/pii/S0168822714001697) [13] | No | Yes, used in online tool and recommended in German practice guideline ([Paprott 2016](https://www.ncbi.nlm.nih.gov/pmc/articles/PMC5128853/pdf/bmjdrc-2016-000280.pdf) [14]) |
| [Rosella (2011)](https://pubmed.ncbi.nlm.nih.gov/20515896/) [15] | DPort | N=19861 | [Kornas (2024)](https://www.ncbi.nlm.nih.gov/pmc/articles/PMC10921488/) [16] | Yes ([Rosella 2014](https://www.sciencedirect.com/science/article/pii/S0091743513003770)) [17] | No | Uptake by public health units and other health settings in Canada ([Kornas 2024](https://www.ncbi.nlm.nih.gov/pmc/articles/PMC10921488/pdf/bmjdrc-2023-003905.pdf) [16]) |
| [Hippisley-Cox (2009)](https://pubmed.ncbi.nlm.nih.gov/19297312/) [18] | QDScore | N=2540753 | [Collins & Altmann (2011)](https://pubmed.ncbi.nlm.nih.gov/21480970/) [19] | Not found | No | No information found |
| [Aekplakorn (2006)](https://pubmed.ncbi.nlm.nih.gov/16873795/) [20] | Thai Diabetes Risk Score | N=2,677 | [Chamnan (2019)](https://diabetesjournals.org/diabetes/article/68/Supplement_1/1515-P/59524/1515-P-Thai-Diabetes-Risk-Score-Performed-Less) [21] | Not found | No | No information found |
| [Kahn (2009)](https://pubmed.ncbi.nlm.nih.gov/19487709/) [22] | Diabetes score based on ARIC study | N=12729 | [Kahn (2009)](https://pubmed.ncbi.nlm.nih.gov/19487709/) [22] | Not found | No | Not found |
| [Ha (2018)](https://pubmed.ncbi.nlm.nih.gov/30113144/) [23] | Korean Diabetes risk score | N=359349 | [Ha (2018)](https://pubmed.ncbi.nlm.nih.gov/30113144/) [23] | Not found | Yes, not mentioned | Not found |
| [Stern (2002)](https://pubmed.ncbi.nlm.nih.gov/11955025/) [24] | San Antonio study prediction model | N=2903 | [Bozorgmanesh (2010)](https://pubmed.ncbi.nlm.nih.gov/20217177/) [25] | Not found | No | Not found |
| [Wilson (2007)](https://pubmed.ncbi.nlm.nih.gov/17533210/) [26] | Framingham Offspring study Type 2 diabetes prediction model | N=3140 | [Lee et al. (2024)](https://www.ncbi.nlm.nih.gov/pmc/articles/PMC10843969/pdf/jkms-39-e47.pdf) [27] | [Lee et al. (2024)](https://www.ncbi.nlm.nih.gov/pmc/articles/PMC10843969/pdf/jkms-39-e47.pdf) [27] | No | [Online calculator](https://www.framinghamheartstudy.org/fhs-risk-functions/diabetes/) [28] |
| [Balkau (2008)](https://pubmed.ncbi.nlm.nih.gov/18689695/) [29] | Prediction score based on DESIR study | N=3817 | [Salinero-Fort (2023)](https://www.medrxiv.org/content/10.1101/2023.11.30.23299228v1.full.pdf+html) [30] | Not found | No | Not found |
| [Amato (2010)](https://pubmed.ncbi.nlm.nih.gov/20067971/) [31] | Visceral Adiposity Index | N=315 + N=1498 | [Amato (2010)](https://pubmed.ncbi.nlm.nih.gov/20067971/) [31] | Not found | No | Not found |
| [Hersberger (2006)](https://pubmed.ncbi.nlm.nih.gov/17004016/) [32] | No name (Sequential screening concept for community pharmacy settings) | N=93258 | Not found | Not found | Yes, not mentioned | Not found |
| [Meigs (2008)](https://www.ncbi.nlm.nih.gov/pmc/articles/PMC2746946/) [33] | Genotype score | N=2377 | Not found | Not found | No | Not found |
| [He (2021)](https://pubmed.ncbi.nlm.nih.gov/33563654/) [34] | Polyexposure score | N=356,621 | [He (2021)](https://pubmed.ncbi.nlm.nih.gov/33563654/) [34] | Not found | Yes, not mentioned | Not found |
| [Pham 2017](https://pubmed.ncbi.nlm.nih.gov/28410981/) [35] | DeepCare | N=7191 | [Pham 2017](https://pubmed.ncbi.nlm.nih.gov/28410981/) [35] | Not found | Yes, not mentioned | Not found |
| [Aasmets (2021)](https://pubmed.ncbi.nlm.nih.gov/33594006/) [36] | No name (Gut microbiome + machine learning) | N=608 | [Aasmets (2021)](https://pubmed.ncbi.nlm.nih.gov/33594006/) [36] | Not found | Yes, not mentioned | Not found |
| NuraLogix (2022) [37] | NuraLogix Corp. (Smartphone app) | N/A | Not found | Not found | No published article | Not found |
| [Cheng (2023)](https://www.nature.com/articles/s43587-023-00391-4) [38] | University of Edinburgh (DNA) | N=14613 | [Cheng (2023)](https://www.nature.com/articles/s43587-023-00391-4) [38] | Not found | Yes, reporting according to TRIPOD | Not found |

| **CKD** |  |  |  |  |  |  |
| --- | --- | --- | --- | --- | --- | --- |
| **Author (year)** | **Prediction approach** | **Sample size** | **Validation** | **Updates** | **Published after Tripod?** | **Applied in practice?** |
| [Hippisley-Cox (2010)](https://pubmed.ncbi.nlm.nih.gov/20565929/) [39] | QKidney score | N=1574749 | [Hippisley-Cox (2010)](https://pubmed.ncbi.nlm.nih.gov/20565929/) [39]  [Collins & Altman (2012)](https://bjgp.org/content/62/597/e243) [40] | Not found | No | Not found, but [online calculator](https://qkidney.org/) [41] available |
| [Alssema (2012)](https://pubmed.ncbi.nlm.nih.gov/22338109/) [42] | Rotterdam-Hoorn score | N=6780 | [Alssema (2012)](https://pubmed.ncbi.nlm.nih.gov/22338109/) [42]  [Rauh (2018)](https://www.ncbi.nlm.nih.gov/pmc/articles/PMC5789113/) [43] | Not found | No | Recommended for use in Dutch practice guideline ([Dekker 2011](https://translational-medicine.biomedcentral.com/articles/10.1186/s12967-020-02434-5#ref-CR18) [44]) |
| [Bang (2007)](https://pubmed.ncbi.nlm.nih.gov/17325299/) [45] | SCORED score | N=8530 | [Bang (2007)](https://pubmed.ncbi.nlm.nih.gov/17325299/) [45]  [Harward (2017)](https://www.ncbi.nlm.nih.gov/pmc/articles/PMC5495467/) | Not found | No | Not found |
| [Chien (2010)](https://pubmed.ncbi.nlm.nih.gov/20800153/) [46] | Clinical model | N=5168 | [Chien (2010)](https://pubmed.ncbi.nlm.nih.gov/20800153/) [46] | Not found | No | Not found |
| [Kshirsagar (2008)](https://pubmed.ncbi.nlm.nih.gov/19064831/) [47] | Simplified categorial model based on ARIC/CHS | N=9470 | [Kshirsagar (2008)](https://pubmed.ncbi.nlm.nih.gov/19064831/) [47] | Not found | No | Not found |
| [Peters (2017)](https://diabetesjournals.org/care/article/40/11/1548/36999/Identification-of-Novel-Circulating-Biomarkers) [48] | PromarkerD | N=345 | [Peters (2019)](https://pubmed.ncbi.nlm.nih.gov/31669066/) [49] | Evaluation by [Fusfeld 2022](https://www.ncbi.nlm.nih.gov/pmc/articles/PMC9342737/) [50] | Yes, not mentioned | PromarkerD-tests can be ordered to test patients <https://promarkerd.com/> [51] |
| [Hu (2020)](https://www.ncbi.nlm.nih.gov/pmc/articles/PMC7346021/) [52] | Dynamic DN incidence nomogram | N=3489 | [Hu (2020)](https://www.ncbi.nlm.nih.gov/pmc/articles/PMC7346021/) [52] | Not found | Yes, not mentioned | [Online Calculator](https://doctorhu.shinyapps.io/DN_DynNomapp/) [53] |
| [Kshirsagar (2008)](https://pubmed.ncbi.nlm.nih.gov/19064831/) [47] | Best fitting categorial model based on ARIC/CHS | N=9470 | [Kshirsagar (2008)](https://pubmed.ncbi.nlm.nih.gov/19064831/) [47] | Not found | No | Not found |
| [Xin (2023)](https://www.frontiersin.org/journals/endocrinology/articles/10.3389/fendo.2023.1108061/full) [54] | HGI risk score | N=1622 | Not found | Not found | Yes, not mentioned | Not found |
| [Chuang (2020)](https://www.researchgate.net/publication/340058121_SUN-156_DNlite-IVD103_a_novel_urinary_test_predicts_progressive_eGFR_decline_in_type_2_diabetes_with_microalbuminuria) [55] | DNlite-IVD103 | N=308 | Not found | Not found | Yes, not mentioned | Not found |
| [Kwon (2012)](https://pubmed.ncbi.nlm.nih.gov/22171932/) [56] | Korean Risk Score | N=6565 | [Kwon (2012)](https://pubmed.ncbi.nlm.nih.gov/22171932/) [56] | Not found | No | Not found |
| [Bradshaw (2019)](https://www.ncbi.nlm.nih.gov/pmc/articles/PMC6730594/) [57] | Point-of-care CKD screening - Model 3a | N=8698 | [Bradshaw (2019)](https://www.ncbi.nlm.nih.gov/pmc/articles/PMC6730594/) [57] | Not found | Yes, not mentioned | Not found |
| [Halbesma (2011)](https://pubmed.ncbi.nlm.nih.gov/21734089/) [58] | Renal risk score based on the PREVEND study | N=8592 | [Halbesma (2011)](https://pubmed.ncbi.nlm.nih.gov/21734089/) [58] | Not found | No | Not found |
| [Li (2018)](https://pubmed.ncbi.nlm.nih.gov/29462347/) [59] | Quality-of-care scoring system | N=4754 | Not found | Not found | Yes, not mentioned | Not found |
| [O'Seaghdha (2012)](https://pubmed.ncbi.nlm.nih.gov/22340925/) [60] | Risk score based on the Framingham Heart Study | N=2490 | [O'Seaghdha (2012)](https://pubmed.ncbi.nlm.nih.gov/22340925/) [60] | Not found | No | Not found |
| [Chien (2010)](https://pubmed.ncbi.nlm.nih.gov/20800153/) [46] | Biochemical model | N=5168 | [Chien (2010)](https://pubmed.ncbi.nlm.nih.gov/20800153/) [46] | Not found | No | Not found |
| [Blech (2011)](https://pubmed.ncbi.nlm.nih.gov/21533139/) [61] | Multifactorial genetic model | N=1274 | [Blech (2011)](https://pubmed.ncbi.nlm.nih.gov/21533139/) [61] | Not found | No | Not found |
| [Liao (2019)](https://pubmed.ncbi.nlm.nih.gov/31882689/) [62] | Genetic risk score based on the GWAS study | N=519 | [Liao (2019)](https://pubmed.ncbi.nlm.nih.gov/31882689/) [62] | Not found | Yes, not mentioned | Not found |
| [Ma (2017)](https://pubmed.ncbi.nlm.nih.gov/28103844/) [63] | Genetic risk score based to on the Framingham Heart Study | N=2698 | Not found | Not found | Yes, not mentioned | Not found |

**References**

1. Conn JW. Interpretation of glucose tolerance test. Am J Med Sci. 1940;199:555.

2. Bogdanet D, O'Shea P, Lyons C, Shafat A, Dunne F. The Oral Glucose Tolerance Test-Is It Time for a Change?-A Literature Review with an Emphasis on Pregnancy. Journal of clinical medicine. 2020;9(11). Epub 2020/10/31. doi: 10.3390/jcm9113451. PubMed PMID: 33121014; PubMed Central PMCID: PMCPMC7693369.

3. De Sanctis V, Soliman A, Daar S, Tzoulis P, Di Maio S, Kattamis C. Oral glucose tolerance test: Ηow to maximize its diagnostic value in children and adolescents. Acta bio-medica : Atenei Parmensis. 2022;93(5):e2022318. Epub 2022/10/28. doi: 10.23750/abm.v93i5.13615. PubMed PMID: 36300215; PubMed Central PMCID: PMCPMC9686143.

4. Peddinti G, Bergman M, Tuomi T, Groop L. 1-Hour Post-OGTT Glucose Improves the Early Prediction of Type 2 Diabetes by Clinical and Metabolic Markers. The Journal of clinical endocrinology and metabolism. 2019;104(4):1131-40. Epub 2018/11/18. doi: 10.1210/jc.2018-01828. PubMed PMID: 30445509; PubMed Central PMCID: PMCPMC6382453.

5. Lindström J, Tuomilehto J. The Diabetes Risk Score: A practical tool to predict type 2 diabetes risk. Diabetes care. 2003;26(3):725-31. doi: 10.2337/diacare.26.3.725.

6. Jølle A, Midthjell K, Holmen J, Carlsen SM, Tuomilehto J, Bjørngaard JH, et al. Validity of the FINDRISC as a prediction tool for diabetes in a contemporary Norwegian population: a 10-year follow-up of the HUNT study. BMJ open diabetes research & care. 2019;7(1):e000769. Epub 2019/12/06. doi: 10.1136/bmjdrc-2019-000769. PubMed PMID: 31803483; PubMed Central PMCID: PMCPMC6887494.

7. Kaczorowski J, Robinson C, Nerenberg K. Development of the CANRISK questionnaire to screen for prediabetes and undiagnosed type 2 diabetes. Can J Diabetes. 2009;33(4):381-5. doi: 10.1016/S1499-2671(09)34008-3.

8. Robinson CA, Agarwal G, Nerenberg K. Validating the CANRISK prognostic model for assessing diabetes risk in Canada's multi-ethnic population. Chronic diseases and injuries in Canada. 2011;32(1):19-31. Epub 2011/12/14. PubMed PMID: 22153173.

9. Canadian Task Force. Diabetes, Type 2 (2012). Guideline Update. 2012 [04.09.2024]. Available from: <https://canadiantaskforce.ca/guidelines/published-guidelines/type-2-diabetes/>.

10. Chen L, Magliano DJ, Balkau B, Colagiuri S, Zimmet PZ, Tonkin AM, et al. AUSDRISK: an Australian Type 2 Diabetes Risk Assessment Tool based on demographic, lifestyle and simple anthropometric measures. Med J Aust. 2010;192(5):274-. doi: <https://doi.org/10.5694/j.1326-5377.2010.tb03507.x>.

11. Buss VH, Varnfield M, Harris M, Barr M. Validation of a lifestyle-based risk score for type 2 diabetes mellitus in Australian adults. Preventive Medicine Reports. 2021;24:101647. doi: <https://doi.org/10.1016/j.pmedr.2021.101647>.

12. Schulze MB, Hoffmann K, Boeing H, Linseisen J, Rohrmann S, Möhlig M, et al. An Accurate Risk Score Based on Anthropometric, Dietary, and Lifestyle Factors to Predict the Development of Type 2 Diabetes. Diabetes care. 2007;30(3):510-5. doi: 10.2337/dc06-2089.

13. Mühlenbruch K, Ludwig T, Jeppesen C, Joost H-G, Rathmann W, Meisinger C, et al. Update of the German Diabetes Risk Score and external validation in the German MONICA/KORA study. Diabetes research and clinical practice. 2014;104(3):459-66. doi: <https://doi.org/10.1016/j.diabres.2014.03.013>.

14. Paprott R, Mühlenbruch K, Mensink GB, Thiele S, Schulze MB, Scheidt-Nave C, et al. Validation of the German Diabetes Risk Score among the general adult population: findings from the German Health Interview and Examination Surveys. BMJ open diabetes research & care. 2016;4(1):e000280. Epub 2016/12/10. doi: 10.1136/bmjdrc-2016-000280. PubMed PMID: 27933187; PubMed Central PMCID: PMCPMC5128853.

15. Rosella LC, Manuel DG, Burchill C, Stukel TA. A population-based risk algorithm for the development of diabetes: development and validation of the Diabetes Population Risk Tool (DPoRT). Journal of epidemiology and community health. 2011;65(7):613-20. Epub 2010/06/03. doi: 10.1136/jech.2009.102244. PubMed PMID: 20515896; PubMed Central PMCID: PMCPMC3112365.

16. Kornas K, Tait C, Negatu E, Rosella LC. External validation and application of the Diabetes Population Risk Tool (DPoRT) for prediction of type 2 diabetes onset in the US population. BMJ open diabetes research & care. 2024;12(2). Epub 2024/03/08. doi: 10.1136/bmjdrc-2023-003905. PubMed PMID: 38453237; PubMed Central PMCID: PMCPMC10921488.

17. Rosella LC, Lebenbaum M, Li Y, Wang J, Manuel DG. Risk distribution and its influence on the population targets for diabetes prevention. Preventive Medicine. 2014;58:17-21. doi: <https://doi.org/10.1016/j.ypmed.2013.10.007>.

18. Hippisley-Cox J, Coupland C, Robson J, Sheikh A, Brindle P. Predicting risk of type 2 diabetes in England and Wales: prospective derivation and validation of QDScore. BMJ. 2009;338:b880. doi: 10.1136/bmj.b880.

19. Collins GS, Altman DG. External validation of QDSCORE(®) for predicting the 10-year risk of developing Type 2 diabetes. Diabetic medicine : a journal of the British Diabetic Association. 2011;28(5):599-607. Epub 2011/04/13. doi: 10.1111/j.1464-5491.2011.03237.x. PubMed PMID: 21480970.

20. Aekplakorn W, Bunnag P, Woodward M, Sritara P, Cheepudomwit S, Yamwong S, et al. A Risk Score for Predicting Incident Diabetes in the Thai Population. Diabetes care. 2006;29(8):1872-7. doi: 10.2337/dc05-2141.

21. Chamnan P, Warasook P, Srisawang O, Phrommachart N, Wettana B, Hcur Investigators. 1515-P: Thai Diabetes Risk Score Performed Less Well in Predicting Incident Diabetes in a General Population: External Validation in the HCUR Cohort. Diabetes. 2019;68(Supplement_1). doi: 10.2337/db19-1515-P.

22. Kahn HS, Cheng YJ, Thompson TJ, Imperatore G, Gregg EW. Two risk-scoring systems for predicting incident diabetes mellitus in U.S. adults age 45 to 64 years. Annals of internal medicine. 2009;150(11):741-51. Epub 2009/06/03. doi: 10.7326/0003-4819-150-11-200906020-00002. PubMed PMID: 19487709.

23. Ha K, Lee Y, Song S, Lee J, Kim D, Cho K, et al. Development and Validation of the Korean Diabetes Risk Score: A 10-Year National Cohort Study. Diabetes Metab J. 2018;42(5):402-14.

24. Stern MP, Williams K, Haffner SM. Identification of persons at high risk for type 2 diabetes mellitus: do we need the oral glucose tolerance test? Annals of internal medicine. 2002;136(8):575-81. Epub 2002/04/17. doi: 10.7326/0003-4819-136-8-200204160-00006. PubMed PMID: 11955025.

25. Bozorgmanesh M, Hadaegh F, Zabetian A, Azizi F. San Antonio heart study diabetes prediction model applicable to a Middle Eastern population? Tehran glucose and lipid study. International journal of public health. 2010;55(4):315-23. Epub 2010/03/11. doi: 10.1007/s00038-010-0130-y. PubMed PMID: 20217177.

26. Wilson PW, Meigs JB, Sullivan L, Fox CS, Nathan DM, D'Agostino RB, Sr. Prediction of incident diabetes mellitus in middle-aged adults: the Framingham Offspring Study. Archives of internal medicine. 2007;167(10):1068-74. Epub 2007/05/30. doi: 10.1001/archinte.167.10.1068. PubMed PMID: 17533210.

27. Lee HA, Park H, Hong YS. Validation of the Framingham Diabetes Risk Model Using Community-Based KoGES Data. Journal of Korean medical science. 2024;39(5):e47. Epub 2024/02/06. doi: 10.3346/jkms.2024.39.e47. PubMed PMID: 38317447; PubMed Central PMCID: PMCPMC10843969.

28. Framingham Heart Study. Diabetes [04.09.2024]. Available from: <https://www.framinghamheartstudy.org/fhs-risk-functions/diabetes/>.

29. Balkau B, Lange Cl, Fezeu L, Tichet J, de Lauzon-Guillain B, Czernichow S, et al. Predicting Diabetes: Clinical, Biological, and Genetic Approaches: Data from the Epidemiological Study on the Insulin Resistance Syndrome (DESIR). Diabetes care. 2008;31(10):2056-61. doi: 10.2337/dc08-0368.

30. Salinero-Fort MA, Mostaza J, Lahoz C, Cárdenas-Valladolid J, Iriarte-Campo V, Estirado-de Cabo E, et al. Validation of Diabetes Prediction Scores: Does adding a high risk for depression increase the area under the curve? medRxiv. 2023:2023.11.30.23299228. doi: 10.1101/2023.11.30.23299228.

31. Amato MC, Giordano C, Galia M, Criscimanna A, Vitabile S, Midiri M, et al. Visceral Adiposity Index: a reliable indicator of visceral fat function associated with cardiometabolic risk. Diabetes care. 2010;33(4):920-2. Epub 2010/01/14. doi: 10.2337/dc09-1825. PubMed PMID: 20067971; PubMed Central PMCID: PMCPMC2845052.

32. Hersberger KE, Botomino A, Mancini M, Bruppacher R. Sequential screening for diabetes--evaluation of a campaign in Swiss community pharmacies. Pharmacy world & science : PWS. 2006;28(3):171-9. Epub 2006/09/28. doi: 10.1007/s11096-006-9034-6. PubMed PMID: 17004016.

33. Meigs JB, Shrader P, Sullivan LM, McAteer JB, Fox CS, Dupuis J, et al. Genotype score in addition to common risk factors for prediction of type 2 diabetes. N Engl J Med. 2008;359(21):2208-19. Epub 2008/11/21. doi: 10.1056/NEJMoa0804742. PubMed PMID: 19020323; PubMed Central PMCID: PMCPMC2746946.

34. He Y, Lakhani CM, Rasooly D, Manrai AK, Tzoulaki I, Patel CJ. Comparisons of Polyexposure, Polygenic, and Clinical Risk Scores in Risk Prediction of Type 2 Diabetes. Diabetes care. 2021;44(4):935-43. doi: 10.2337/dc20-2049.

35. Pham T, Tran T, Phung D, Venkatesh S. Predicting healthcare trajectories from medical records: A deep learning approach. J Biomed Inform. 2017;69:218-29. doi: <https://doi.org/10.1016/j.jbi.2017.04.001>.

36. Aasmets O, Lüll K, Lang JM, Pan C, Kuusisto J, Fischer K, et al. Machine Learning Reveals Time-Varying Microbial Predictors with Complex Effects on Glucose Regulation. mSystems. 2021;6(1). Epub 2021/02/18. doi: 10.1128/mSystems.01191-20. PubMed PMID: 33594006; PubMed Central PMCID: PMCPMC8573957.

37. Webb M. HLTH 2022: NuraLogix Demo AI Models That Can Predict Risk For Pre-Diabetes: Medtech Insight; 2022 [21.06.2023]. Available from: <https://medtech.pharmaintelligence.informa.com/MT146039/HLTH-2022-NuraLogix-Demo-AI-Models-That-Can-Predict-Risk-For-Pre-Diabetes>.

38. Cheng Y, Gadd DA, Gieger C, Monterrubio-Gómez K, Zhang Y, Berta I, et al. Development and validation of DNA methylation scores in two European cohorts augment 10-year risk prediction of type 2 diabetes. Nat Aging. 2023;3(4):450-8. doi: 10.1038/s43587-023-00391-4.

39. Hippisley-Cox J, Coupland C. Predicting the risk of Chronic Kidney Disease in Men and Women in England and Wales: prospective derivation and external validation of the QKidney®Scores. BMC Fam Pract. 2010;11(1):49. doi: 10.1186/1471-2296-11-49.

40. Collins GS, Altman DG. Predicting the risk of chronic kidney disease in the UK: an evaluation of QKidney® scores using a primary care database. The British journal of general practice : the journal of the Royal College of General Practitioners. 2012;62(597):e243-50. Epub 2012/04/24. doi: 10.3399/bjgp12X636065. PubMed PMID: 22520911; PubMed Central PMCID: PMCPMC3310030.

41. ClinRisk Ltd. Welcome to the QKidney®-2018 risk calculator. 2019 [04.09.2024]. Available from: <https://qkidney.org/>.

42. Alssema M, Newson RS, Bakker SJ, Stehouwer CD, Heymans MW, Nijpels G, et al. One risk assessment tool for cardiovascular disease, type 2 diabetes, and chronic kidney disease. Diabetes care. 2012;35(4):741-8. Epub 2012/02/18. doi: 10.2337/dc11-1417. PubMed PMID: 22338109; PubMed Central PMCID: PMCPMC3308277.

43. Rauh SP, Rutters F, van der Heijden A, Luimes T, Alssema M, Heymans MW, et al. External Validation of a Tool Predicting 7-Year Risk of Developing Cardiovascular Disease, Type 2 Diabetes or Chronic Kidney Disease. Journal of general internal medicine. 2018;33(2):182-8. Epub 2017/12/06. doi: 10.1007/s11606-017-4231-7. PubMed PMID: 29204973; PubMed Central PMCID: PMCPMC5789113.

44. Dekker JM, Alssema M, Janssen P, Paardl M, Festen CCS, Oosterhout MJW, et al. Guideline prevention of cardiometabolic diseases. Huisarts en Wetenschap. 2011;54:138-55.

45. Bang H, Vupputuri S, Shoham DA, Klemmer PJ, Falk RJ, Mazumdar M, et al. SCreening for Occult REnal Disease (SCORED): a simple prediction model for chronic kidney disease. Archives of internal medicine. 2007;167(4):374-81. Epub 2007/02/28. doi: 10.1001/archinte.167.4.374. PubMed PMID: 17325299.

46. Chien KL, Lin HJ, Lee BC, Hsu HC, Lee YT, Chen MF. A prediction model for the risk of incident chronic kidney disease. The American journal of medicine. 2010;123(9):836-46.e2. Epub 2010/08/31. doi: 10.1016/j.amjmed.2010.05.010. PubMed PMID: 20800153.

47. Kshirsagar AV, Bang H, Bomback AS, Vupputuri S, Shoham DA, Kern LM, et al. A simple algorithm to predict incident kidney disease. Archives of internal medicine. 2008;168(22):2466-73. Epub 2008/12/10. doi: 10.1001/archinte.168.22.2466. PubMed PMID: 19064831; PubMed Central PMCID: PMCPMC2849985.

48. Peters KE, Davis WA, Ito J, Winfield K, Stoll T, Bringans SD, et al. Identification of Novel Circulating Biomarkers Predicting Rapid Decline in Renal Function in Type 2 Diabetes: The Fremantle Diabetes Study Phase II. Diabetes care. 2017;40(11):1548-55. doi: 10.2337/dc17-0911.

49. Peters KE, Davis WA, Ito J, Bringans SD, Lipscombe RJ, Davis TME. Validation of a protein biomarker test for predicting renal decline in type 2 diabetes: The Fremantle Diabetes Study Phase II. Journal of diabetes and its complications. 2019;33(12):107406. Epub 2019/11/02. doi: 10.1016/j.jdiacomp.2019.07.003. PubMed PMID: 31669066.

50. Fusfeld L, Murphy JT, Yoon Y, Kam LY, Peters KE, Lin Tan P, et al. Evaluation of the clinical utility of the PromarkerD in-vitro test in predicting diabetic kidney disease and rapid renal decline through a conjoint analysis. PloS one. 2022;17(8):e0271740. Epub 2022/08/02. doi: 10.1371/journal.pone.0271740. PubMed PMID: 35913946; PubMed Central PMCID: PMCPMC9342737.

51. Proteomics International. PromarkerD. 2017 [04.09.2024]. Available from: <https://promarkerd.com/>.

52. Hu Y, Shi R, Mo R, Hu F. Nomogram for the prediction of diabetic nephropathy risk among patients with type 2 diabetes mellitus based on a questionnaire and biochemical indicators: a retrospective study. Aging. 2020;12(11):10317-36. Epub 2020/06/03. doi: 10.18632/aging.103259. PubMed PMID: 32484786; PubMed Central PMCID: PMCPMC7346021.

53. shinyapps.io. DN_Dynamic Nomogram 2022 [04.09.2024]. Available from: <https://doctorhu.shinyapps.io/DN_DynNomapp/>.

54. Xin S, Zhao X, Ding J, Zhang X. Association between hemoglobin glycation index and diabetic kidney disease in type 2 diabetes mellitus in China: A cross- sectional inpatient study. Frontiers in endocrinology. 2023;14:1108061. Epub 2023/03/28. doi: 10.3389/fendo.2023.1108061. PubMed PMID: 36967789; PubMed Central PMCID: PMCPMC10031087.

55. Chuang LM, Lin WY, Huang CH, Lin CH, Tseng TL. SUN-156 DNlite-IVD103, a novel urinary test, predicts progressive eGFR decline in type 2 diabetes with microalbuminuria. Kidney Int Rep. 2020;5:S264. doi: 10.1016/j.ekir.2020.02.685.

56. Kwon KS, Bang H, Bomback AS, Koh DH, Yum JH, Lee JH, et al. A simple prediction score for kidney disease in the Korean population. Nephrology (Carlton, Vic). 2012;17(3):278-84. Epub 2011/12/17. doi: 10.1111/j.1440-1797.2011.01552.x. PubMed PMID: 22171932.

57. Bradshaw C, Dimple K, Montez-Rath ME, Han J, Zheng Y, Shivashankar R, et al. Early detection of chronic kidney disease in low-income and middle-income countries: development and validation of a point-of-care screening strategy for India. BMJ Global Health. 2019;4(5):e001644. doi: 10.1136/bmjgh-2019-001644.

58. Halbesma N, Jansen DF, Heymans MW, Stolk RP, de Jong PE, Gansevoort RT. Development and validation of a general population renal risk score. Clinical journal of the American Society of Nephrology : CJASN. 2011;6(7):1731-8. Epub 2011/07/08. doi: 10.2215/cjn.08590910. PubMed PMID: 21734089.

59. Li PI, Wang JN, Guo HR. Long-term quality-of-care score predicts incident chronic kidney disease in patients with type 2 diabetes. Nephrology, dialysis, transplantation : official publication of the European Dialysis and Transplant Association - European Renal Association. 2018;33(11):2012-9. Epub 2018/02/21. doi: 10.1093/ndt/gfx375. PubMed PMID: 29462347.

60. O'Seaghdha CM, Lyass A, Massaro JM, Meigs JB, Coresh J, D'Agostino RB, Sr., et al. A risk score for chronic kidney disease in the general population. The American journal of medicine. 2012;125(3):270-7. Epub 2012/02/22. doi: 10.1016/j.amjmed.2011.09.009. PubMed PMID: 22340925; PubMed Central PMCID: PMCPMC3285426.

61. Blech I, Katzenellenbogen M, Katzenellenbogen A, Wainstein J, Rubinstein A, Harman-Boehm I, et al. Predicting diabetic nephropathy using a multifactorial genetic model. PloS one. 2011;6(4):e18743. Epub 2011/05/03. doi: 10.1371/journal.pone.0018743. PubMed PMID: 21533139; PubMed Central PMCID: PMCPMC3077408.

62. Liao LN, Li TC, Li CI, Liu CS, Lin WY, Lin CH, et al. Genetic risk score for risk prediction of diabetic nephropathy in Han Chinese type 2 diabetes patients. Sci Rep. 2019;9(1):19897. Epub 2019/12/29. doi: 10.1038/s41598-019-56400-3. PubMed PMID: 31882689; PubMed Central PMCID: PMCPMC6934611.

63. Ma J, Yang Q, Hwang SJ, Fox CS, Chu AY. Genetic risk score and risk of stage 3 chronic kidney disease. BMC nephrology. 2017;18(1):32. Epub 2017/01/21. doi: 10.1186/s12882-017-0439-3. PubMed PMID: 28103844; PubMed Central PMCID: PMCPMC5248454.
